# Elucidating Conserved Transcriptional Networks Underlying Pesticide Exposure and Parkinson's Disease: A Focus on Chemicals of Epidemiological Relevance

**DOI:** 10.3389/fgene.2018.00701

**Published:** 2019-01-25

**Authors:** Fangjie Cao, Christopher L. Souders II, Veronica Perez-Rodriguez, Christopher J. Martyniuk

**Affiliations:** Department of Physiological Sciences, Center for Environmental and Human Toxicology, University of Florida Genetics Institute, College of Veterinary Medicine, University of Florida Interdisciplinary Program in Biomedical Sciences Neuroscience, University of Florida, Gainesville, FL, United States

**Keywords:** neurodegeneration, pesticides, mitochondria, gene networks, adverse outcome pathways

## Abstract

While a number of genetic mutations are associated with Parkinson's disease (PD), it is also widely acknowledged that the environment plays a significant role in the etiology of neurodegenerative diseases. Epidemiological evidence suggests that occupational exposure to pesticides (e.g., dieldrin, paraquat, rotenone, maneb, and ziram) is associated with a higher risk of developing PD in susceptible populations. Within dopaminergic neurons, environmental chemicals can have an array of adverse effects resulting in cell death, such as aberrant redox cycling and oxidative damage, mitochondrial dysfunction, unfolded protein response, ubiquitin-proteome system dysfunction, neuroinflammation, and metabolic disruption. More recently, our understanding of how pesticides affect cells of the central nervous system has been strengthened by computational biology. New insight has been gained about transcriptional and proteomic networks, and the metabolic pathways perturbed by pesticides. These networks and cell signaling pathways constitute potential therapeutic targets for intervention to slow or mitigate neurodegenerative diseases. Here we review the epidemiological evidence that supports a role for specific pesticides in the etiology of PD and identify molecular profiles amongst these pesticides that may contribute to the disease. Using the Comparative Toxicogenomics Database, these transcripts were compared to those regulated by the PD-associated neurotoxicant MPTP (1-methyl-4-phenyl-1,2,3,6-tetrahydropyridine). While many transcripts are already established as those related to PD (alpha-synuclein, caspases, leucine rich repeat kinase 2, and parkin2), lesser studied targets have emerged as “pesticide/PD-associated transcripts” [e.g., phosphatidylinositol glycan anchor biosynthesis class C (Pigc), allograft inflammatory factor 1 (Aif1), TIMP metallopeptidase inhibitor 3, and DNA damage inducible transcript 4]. We also compared pesticide-regulated genes to a recent meta-analysis of genome-wide association studies in PD which revealed new genetic mutant alleles; the pesticides under review regulated the expression of many of these genes (e.g., ELOVL fatty acid elongase 7, ATPase H+ transporting V0 subunit a1, and bridging integrator 3). The significance is that these proteins may contribute to pesticide-related increases in PD risk. This review collates information on transcriptome responses to PD-associated pesticides to develop a mechanistic framework for quantifying PD risk with exposures.

## Introduction

Neurodegeneration is underscored by a series of sequential events that occur in a complex fashion; these process are due to a myriad of genetic, physiological, and environmental factors that can occur over time. As such, knowledge as to the mechanisms underlying these events is critical for therapeutic interventions and to abate human risk. One devastating neurodegenerative disease is Parkinson's disease (PD), first observed in the early 1800's by London doctor James Parkinson and which was subsequently described in essays about the “shaking palsy” disorder (Parkinson, 2002). PD is a neurodegenerative disease and movement disorder characterized by the loss of dopaminergic neurons in the substantia nigra, the clinical signs of which include tremors, impaired fine motor dexterity, changes in gait, impaired gross motor coordination, stiffness, and increased muscle tone. PD involves genetic variants and/or damage (neurotoxicity) that leads to the subsequent loss of dopaminergic neurons and dopamine production over time. While risk factors for the disease are many (genetics, age, sex, and diet) (Emamzadeh and Surguchov, 2018), there is also a significant environmental component which includes occupational exposures to pesticides. In the USA, PD affects an estimated 2% of Americans aged ≥60 years (Alves et al., 2008) and costs Medicare in excess of $23 billion annually (Huse et al., 2005). Moreover, the number of individuals with PD over the age of 50 is expected to double between 8.7 and 9.3 million by 2030 in many developed countries (Dorsey et al., 2007). It is estimated that ~20% PD is explainable via a genetic basis (Bekris et al., 2010), and although both genetics and age are major factors in the disease, it has been clear for some time that our environment plays a significant role in the etiology of PD (Betarbet et al., 2000).

Pesticide exposure is associated with a myriad of human neurological diseases, including PD (Freire and Koifman, 2012; Martenies and Perry, 2013; Starling et al., 2014). It should be noted however, that there is some debate about the precise role of pesticide exposures in disease etiology. For example, while evidence supports a generic association between pesticide exposure and PD, a direct causal link for many pesticides is difficult to ascertain due to gaps in epidemiological study design (Ascherio et al., 2006; Brown et al., 2006). Despite some unanswered questions, experimental evidence for pesticide induced neurodegeneration remains convincing for select chemicals. For example, experimental data illustrating a link between the herbicide paraquat and PD is quite compelling in that this herbicide plays a direct role in dopamine cell death as it is actively taken into the cell by dopamine active transporters (Rappold et al., 2011). It therefore remains important to quantify risks associated with acute and continuous pesticide exposure.

The precise mechanisms underlying the relationship between neuroactive pesticides and pathological conditions associated with neurodegenerative disease are many and include aberrant redox cycling and oxidative damage, mitochondrial dysfunction, ATP deficits, impaired unfolded protein response, neuroinflammation, and metabolic disruption among others (Sun et al., 2005; Castello et al., 2007; Hatcher et al., 2007; Cristóvão et al., 2009; Franco et al., 2010; Roede et al., 2011; Lei et al., 2014; Caito and Aschner, 2015; Cowie et al., 2017a,b). Mitochondrial dysfunction is well documented to contribute to dopaminergic cell death and PD pathology (Camilleri and Vassallo, 2014; Huang et al., 2016; Helley et al., 2017; Schmidt et al., 2017) and studies have identified specific genetic correlates (i.e., PARK genes) associated with the etiopathogenetic nature of the disease (reviewed in Helley et al., 2017). Understanding the role of specific signaling pathways under low, chronic pesticide exposures (i.e., exposome) is an important facet for understanding how our environment contributes to PD and other neurodegenerative diseases. Critical to this are studies designed to identify (1) compensatory mechanisms that are activated in dopamine neurons following exposure to neuro-toxicants; (2) signaling pathways that are conserved among chemicals that lead to the same adverse effect, for example mitochondrial dysfunction and dopamine cell death. These signaling pathways represent universal targets for therapeutic interventions. Indeed, adverse outcome pathways (AOPs) are currently under development for neurodegenerative diseases such as PD (Bal-Price and Meek, 2017; Terron et al., 2018) and mechanistic insight is needed to further inform and annotate these AOPs with early molecular initiating events.

**Figure 1:**
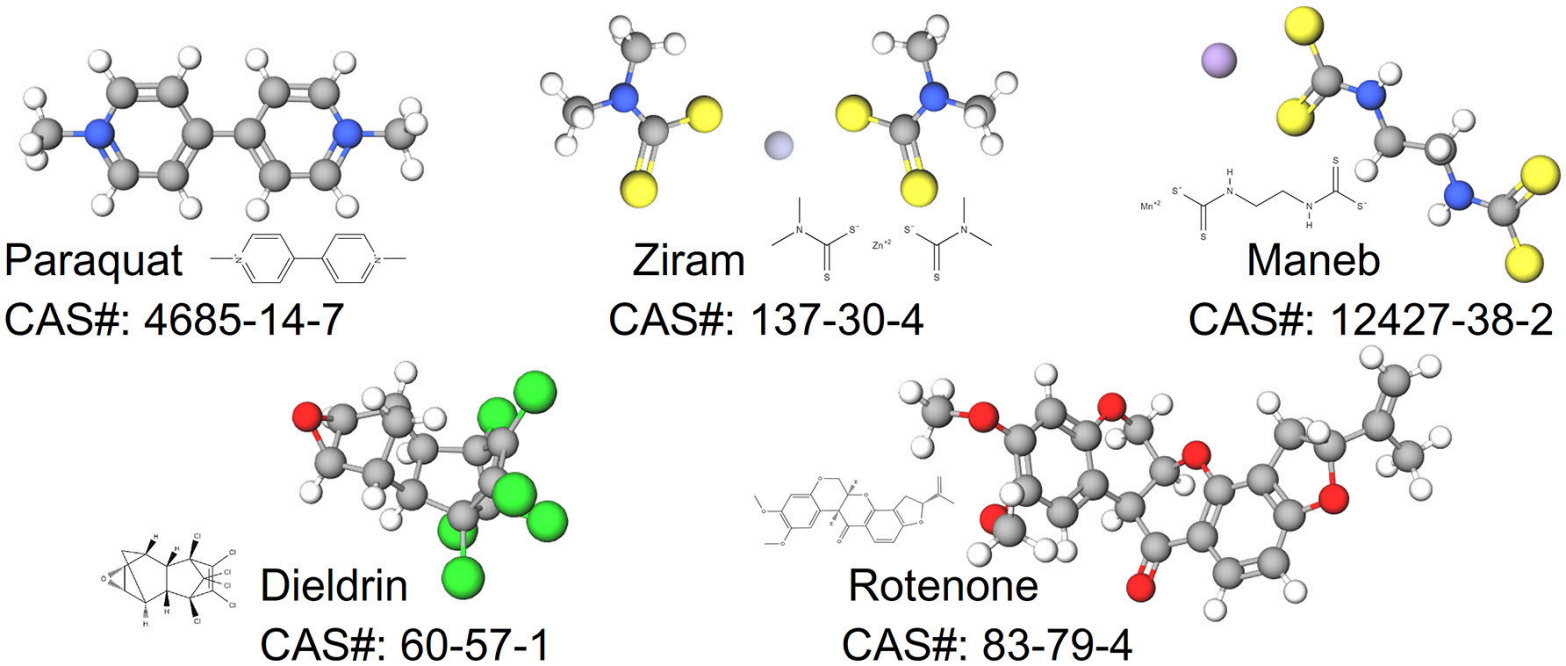
Structures for pesticides epidemiologically associated with Parkinson's disease.

In this review, we synthesize the current state of knowledge for transcriptional responses to select pesticides linked to PD. We focus on pesticides for which there is relatively strong epidemiological evidence for an association between exposure and increased risk to PD (dieldrin, paraquat, rotenone, and the dithiocarbamates, which are represented here by maneb and ziram). The structures of these chemicals are shown in Figure 1. This review is not meant to be comprehensive and will not cover all known mechanisms, nor does it include all pesticides suspected of being associated to PD. Rather the approach is to first focus on a smaller, but diverse group of pesticides with relatively strong epidemiological evidence. For these pesticides, we review studies that reveal mechanisms related to adverse outcomes from pesticide exposures. Advances in computational biology are generating new opportunities for elucidating the complex nature of neurodegenerative diseases including PD (Rakshit et al., 2014; Glaab and Schneider, 2015). Transcriptome profiling has uncovered novel signaling pathways to experimentally interrogate in PD models (Gollamudi et al., 2012) and genetics and metabolomics studies are generating new knowledge about adverse effects in models of PD (Roede et al., 2014). Molecular networks can inform these adverse outcome pathways, improving intervention strategies for neurotoxicity. To better synthesize information, we leveraged the Comparative Toxicogenomics Database (Davis et al., 2009, 2016) and Pathway Studio (Elsevier) to describe expression patterns on a global scale. One long term goal for such integration is to create a pesticide-specific exposome to improve risk characterization and to generate a framework from which to compare new pesticides. We begin with a brief description of the pesticides (Table 1), information on their historical use, and review of studies that investigate the mechanisms involved in the chemical neurotoxicity. Some of these pesticides have been discussed previously for their role in PD etiology (Hatcher et al., 2008; Helley et al., 2017) and here we focus on the transcriptional responses following exposure. These pesticides to date represent some of the most likely candidates with epidemiological links to PD (Table 2). The purpose of this review is to elucidate new therapeutic targets and pathways for PD by computationally interrogating pesticides that are linked to the disease.

**Table 1:** Summary of select pesticides associated with Parkinson's disease.

**Pesticide**	**Type**	**Major Mode of Action**
Dieldrin	Insecticide	An organochlorine pesticide that is highly persistent, showing high bioaccumulation potential in lipid rich tissues such as the brain. The primary mechanism of neurotoxicity is antagonism of the GABA_A_ receptor, preventing influx of chloride into the neuron and leading to excitation and death due to a lack of neuronal inhibition. This pesticide was banned in many developed countries in the 1970s but remains detectable in some environmental samples, as well as animal and human tissues.
Paraquat	Herbicide	Non-selective herbicide which is readily absorbed by plants. Paraquat impairs photosynthetic complexes and disrupts cell membranes due to its high propensity for redox cycling. Paraquat can cross the blood brain barrier (BBB) in a non-destructive, Na+, dependent manner in animal models. Paraquat is imported into dopaminergic neurons via dopamine active transporters, a mechanism similar to the neurotoxin MPTP. Both paraquat and MPTP are similar in chemical structure. Paraquat poisoning also leads to pulmonary toxicity or “Paraquat lung”.
Rotenone	Insecticide	A selective, but non-specific insecticide widely used in home gardens. It induces toxicity by inhibiting Complex I in the electron transporting chain of the mitochondria, showing relatively high toxicity in the nervous system of animals. Neuronal tissue exposed to rotenone showed high oxidative damage and this can lead to a loss of dopamine neurons.
Maneb	Fungicide	Maneb is a manganese-containing ethylenebis-dithiocarbamate fungicide used as a pesticide on fruit and a variety of other crops. Epidemiological studies have indicated that exposure to maneb alone or co-exposure to other pesticides increase the risk of Parkinsonism-like syndrome. This chemical can inhibit oxidative respiration via complex III inhibition in cells.
Ziram	Fungicide	Similar to maneb, this pesticide is a dithiocarbamate but is a member of the dimethyl-dithiocarbamates (DMDC) family. Individuals exposed to both maneb and ziram showed a 3-fold increase in the risk to PD. Compared to maneb, much less is known about the mechanisms underlying this pesticide.

**Table 2:** Examples of clinical and epidemiological evidence supporting a link between pesticides under review and Parkinson's disease.

**Type of Study**	**Location**	**Year Published**	**Subjects**	**Results - Relative Risk or Odds ratio (95% CI)**	**Reference**
**DIELDRIN**					
Case-Control Study	Finland	2010	101 PD cases, 349 matched controls	Interquartile range, 1.95 (1.26–3.02) *p* = 0.003; Confirmed cases, never smoked	Weisskopf et al., 2010
Case-Control Study	India	2013	145 subjects in the age group of 50 to 85 years, 70 subjects diagnosed with PD were enrolled	2.09 (1.41-3.11), *p* < 0.001	Chhillar et al., 2013
**PARAQUAT**					
Case-Control Study	Canada	1990	57 PD cases reported from physicians in the area, 122 age-matched controls randomly selected from electoral rolls	4 ca, 0 co Expose to paraquat *p* < 0.01	Hertzman et al., 1990
Case-Control Study	Canada	1994	127 PD cases reported from physicians in area; 245 Controls randomly chosen from electoral rolls; 121 Patients with cardiac disease (CD)	1.25 (0.34, 4.63), Population 1.11 (0.32, 3.87), CD	Hertzman et al., 1994
Case-Control Study	Germany	1996	380 PD cases aged 65 or less; 379 Neighborhood controls	1 ca, 0 co exposed to paraquat	Seidler et al., 1996
Case-Control Study	Taiwan	1997	376 regional controls, 120 PD cases, 240 controls from the same hospital	3.22 (2.41, 4.31) exposed to paraquat, 6.44 (2.41, 17.2) 20+ years of use	Liou et al., 1997
Case-Control Study	Finland	1999	123 PD cases, 246 matched controls	3 cases and 5 controls reported the use of paraquat	Kuopio et al., 1999
Cohort Study	USA	2001	310 subjects selected and examined neurologically	0.8 (0.5, 1.3) with any paraquat exposure; 0.9 (0.4, 2.4) Highest tertile exposure; 0.7 (0.5, 1.9) highest acre-years	Engel et al., 2001
Case-Control Study	USA	2005	100 cases from a private neurology practice, 84 controls from that same practice	3.2 (0.4, 31.6)	Firestone et al., 2005
Cohort Study	USA	2007	83 Prevalent cases, 78 incident, 79557 without PD	1.8 (1.0, 3.4) in prevalent cases; 1.0 (0.5, 1.9) in incident cases	Kamel et al., 2007
Case-Control Study	USA	2008	250 cases, 388 controls	1.67 (0.22, 12.76)	Dhillon et al., 2008
Case-Control Study	France	2009	224 cases, 557 matched controls from the French health insurance system for agricultural workers	1.2 (0.7, 2.1) all men; 1.6 (0.7, 3.4) Men age 65+	Elbaz et al., 2009
Case-Control Study	USA	2009	368 cases, 31 randomly selected controls	1.26 (0.72, 2.20) well water, 1.15 (0.82, 1.62) Ambient alone, 1.19 (0.77, 1.82) ambient or well water	Gatto et al., 2009
Case-Control Study	USA	2009	368 Cases, 346 Controls	1.01 (0.71, 1.42) paraquat alone, 1.75 (1.13, 2.73) paraquat+maneb	Costello et al., 2009
Case-Control Study	USA	2009	324 cases, 334 controls	2.99 (0.88, 3.48) Maneb+paraquat in those with 1 susceptible allele. 4.53 (1.70, 12.09) maneb + paraquat in those with 2+ susceptible alleles	Ritz et al., 2009
Case-Control Study	North America	2009	519 cases, 511 controls	2.80 (0.81, 9.72)	Tanner et al., 2009
Case-Control Study	USA	2011	110 cases, 358 controls	2.5 (1.4, 4.7); 2.4 (1.0, 5.5) < median duration; 3.6 (1.6, 8.1) > median duration	Tanner et al., 2011
Case-Control Study	USA	2011	362 Cases from neurology practices, 341 controls from Medicare records and randomly selected	1.26 (0.86, 1.86) paraquat alone; 1.82 (1.03, 321) paraquat + ziram; 3.09 (1.69, 5.64) paraquat + ziram + maneb	Wang et al., 2011
Case-Control Study	USA	2012	404 cases, 526 controls	0.90 (0.14, 5.43)	Firestone et al., 2010
**ROTENONE**					
Case-Control Study	USA	2007	83 prevalent cases and 79,557 controls.	1.7 (0.6–4.7) with past rotenone use.	Kamel et al., 2007
Case-Control Study	USA	2008	100 cases and 84 controls	10.0 (2.9–34.3) with use of “organic pesticides such as rotenone”	Dhillon et al., 2008
Case-Control Study	USA	2008	319 cases and 296 relative and other controls	5.93 (0.63–56.10) with Botanical insecticide class including rotenone	Hancock et al., 2008
Case-Control Study	USA	2009	519 cases and 511 controls	0.82 (0.05–13.34) with past rotenone use. (No association with PD)	Tanner et al., 2009
Case-Control Study	USA	2011	110 PD cases and 358 controls	OR = 2.5; 95% CI, 1.3–4.7 with past exposure to rotenone	Tanner et al., 2011
Case-Control Study	USA	2015	69 cases and 237 controls	OR = 3.7 (1.7, 8.1), 3.8 (1.5, 9.6), 5.5 (2.0, 15.3) with exposure to rotenone (*P* < 0.01)	Furlong et al., 2015
Cohort Study	France	2017	181,842 agricultural workers (4916 exposed to Rotenone)	1.57 (1.08, 2.29), with past exposure to rotenone	Pouchieu et al., 2017
**MANEB**					
Case Study	Brazil	1988	Two men of PD case	Two men presented parkinsonian syndromes and parkinsonian syndromes, moreover, significantly higher prevalence of parkinsonian syndromes were observed in fifty male rural workers with occupational exposure to maneb	Ferraz et al., 1988
Case Study	Italy	1994	One man PD case	A man was exposed to maneb at 35-37-old age, a mild tremor associated with paresthesia appeared in his right leg and later spread to the ipsilateral arm, moreover, these symptoms then remained stable for about seven years.	Meco et al., 1994
Case-Control Study	USA	2009	324 incident PD patients, 334 controls	2.99 (0.88, 3.48) in those with 1 susceptible allele 4.53 (1.70, 12.09) in those with 2 + susceptible alleles	Ritz et al., 2009
Case-Control Study	USA	2009	368 incident PD cases, 341 controls	1.75 (1.13, 2.73)	Costello et al., 2009
Case-Control Study	USA	2011	362 Cases from neurology practices; 341 controls from Medicare records	3.09 (1.69, 5.64) when exposed to paraquat + ziram + maneb	Wang et al., 2011
Case-Control Study	USA	2014	953 cases and 816 controls	1.59 (0.92–2.67) at residence only, 1.92 (1.06–3.10) at workplace only, and 5.07 (1.58–12.6) at both conditions	Fitzmaurice et al., 2014
Case-Control Study	Netherlands	2017	202 PD cases and 362 hospital-based controls	0.97 (0.72–1.30)	Brouwer et al., 2017
**ZIRAM**					
Case-Control Study	USA	2011	362 Cases from neurology practices; 341 controls from Medicare records	1.82 (1.03, 321) paraquat + ziram; 3.09 (1.69, 5.64) paraquat + ziram + maneb; 5.98 (1.95, 18.32) in younger onset patients that were exposed to a combination of ziram and paraquat	Wang et al., 2011
Case-Control Study	USA	2013	360 PD cases and 816 population-based controls	3.30 (1.50, 7.26) when exposed to ziram at both residence and workplace	Rhodes et al., 2013
Case-Control Study	USA	2014	953 cases and 816 controls	3.33 (1.62, 6.88) when exposed to ziram at both residence and workplace	Fitzmaurice et al., 2014

## Pesticides

### The Organochlorine Pesticides: the Case Study of Dieldrin

#### Brief History of Use

Organochlorine pesticides (OCPs) were among some of the first synthetically designed pesticides in the late 1940s-50s. These chlorinated organic chemicals included dichlorodiphenyltrichloroethane (DDT) and a number of its derivatives p′-DDD, p,p′-DDT, p,p′-DDE. Other types of OCPs that were used heavily in agriculture in the 1950–70s included dicofol, chlordane, heptaclor, endosufan, and toxaphene. In addition to these OCPs, two other popular cyclodienes (chlorinated polycyclic pesticides derived from cyclopentadiene), aldrin and dieldrin, were used extensively in corn, potato, and cotton agriculture at the time. These two pesticides are structurally very similar, the only difference being that dieldrin has an epoxied ring at the site of one of the carbon-carbon double bonds present in aldrin. Noteworthy is that aldrin is rapidly converted to dieldrin under environmental conditions (Metcalf et al., 1973), thus dieldrin is considered to be more of an environmental and human health concern than aldrin. OCPs were highly effective broad spectrum pesticides, however it became apparent over time that these chemicals were extremely persistent, highly toxic to non-target organisms (birds, fish), and bio-accumulated in lipid-rich tissues (Jorgenson, 2001). As such, many of these pesticides were increasingly regulated and restricted in the 1970s by governmental agencies such as the Environmental Protection Agency. Indeed the Stockholm Convention on Persistent Organic Pollutants, an international environmental treaty signed in 2001, aimed to eliminate the production and use of persistent organic pollutants. However, despite these efforts, pesticides belonging to the class of OCPs are still used in developing countries because they are cost effective and potent for pest control (Jayaraj et al., 2016). In fact, aldrin and dieldrin continued for many years to be among the most widely used pesticides in some developing countries (Gupta, 2004). Due to their persistence, propensity to bioaccumulate in organisms, and relative abundance globally, OCPs remain an environmental concern. In 2017, the U.S. Agency for Toxic Substances and Disease Registry ranked on the Substance Priority List many OCPs in the top 50 of concern: *p,p*′-DDT (#13), *p,p*′-DDE (#21), chlordane (#22), *p,p*′-DDD (#26), toxaphene (#32) endrin (#40), and endosulfan (#44). Dieldrin (#18) and aldrin (#25) also were among the top 50 chemicals.

#### Mechanism of Action

One of the primary mechanisms of action of dieldrin is to bind to, and block the gamma-aminobutyric acid A (GABA_A_) receptor-chloride channel complex (Nagata and Narahashi, 1994). GABA is an inhibitory neurotransmitter in the central nervous system and opens GABA_A_ channels to chloride in order to hyperpolarize cells. Dysfunction in these receptors result in over-excitation, seizures, and death. Studies have shown some activity as a mitochondrial toxicant (Schmidt et al., 2017) and endocrine disruptor, and dieldrin has been reported to disrupt the mitochondrial electron transport chain (Pardini et al., 1971) and to act as weak estrogen receptor agonists and antagonists (Andersen et al., 2002) in estrogen and androgen transactivation assays. Detailed structure-activity relationships have also been investigated for dieldrin in terms of cellular interactions and dieldrin was found to disrupt a wide number of cellular processes including the dopamine synthesis pathway that were dependent upon both its stereochemistry and structure (Allen et al., 2013). Thus, while dieldrin is a potent GABA_A_ receptor antagonist, the modes of action for dieldrin are diverse in the CNS.

#### Epidemiological Evidence

Dieldrin is a long-lived pesticide that accumulates in lipid rich tissues. Over the past several decades, studies have indicated potential associations between dieldrin exposure and increased risk to PD (Table 2). Studies in the postmortem human brain have found that concentrations of dieldrin can be significantly higher in PD patients compared with individuals who are not diagnosed with PD (Fleming et al., 1994; Corrigan et al., 2000). Weisskopf et al. (2010) conducted a nested case-control study using serum collected from Finnish mobile clinic health examination surveys. The serum was collected in 1968–72, and it was assessed ~35 years later for pesticide levels. Levels were then compared to the incidence of PD (odds ratio) following adjustments for sex, age, and vital status among other variables. Dieldrin was the only pesticide measured in the blood that showed a positive association with an increased incidence of PD with a 1.95 odds ratio (*p* = 0.003) (Weisskopf et al., 2010). This was the case when considering non-smokers, as smoking offers some protection against developing PD (Morens et al., 1995). In another study in India, a case control study included 145 subjects in the age group of 50–85 years and 70 subjects were diagnosed with PD (Chhillar et al., 2013). The control group was comprised of age-matched healthy volunteers and a number of OCPs were assessed from the blood. Dieldrin was detectable in the control group in 9.3% of the cases, however for those with Parkinson's disease, dieldrin was detected in 61.4% of the patients. Levels of dieldrin ranged from 2- to a 6-fold increase in the PD group compared to individuals in the control group. Thus, epidemiological studies support an association between dieldrin and PD.

#### Cellular and Molecular Responses

Dieldrin has been shown to negatively affect both cells and animal models within the context of the dopamine system. In cell models used to study dopamine release such as the PC12 line (derived from pheochromocytoma of the rat adrenal medulla), dieldrin-treated cells induced reactive oxygen species and apoptosis at doses ranging from 30 to 1000 μM after 1 h (Kitazawa et al., 2001). These events were also accompanied by increased DNA fragmentation in PC12 cells. In another study, dieldrin between 5 and 25 μM has been shown to affect endpoints related to mitochondrial dysfunction and apoptosis in rat dopaminergic cell lines in combination with other organochlorines (Sharma et al., 2009). More recently, a study exposing dopaminergic rat cells (N27) to 25 μM dieldrin for 24 h revealed a 40–50% reduction in oxygen consumption rates of cells following the addition of mitochondrial toxicants into the culture, further evidence that dieldrin impairs mitochondrial bioenergetics in dopamine cells (Schmidt et al., 2017). Noteworthy was that this response was also associated with a 3-fold induction of transcript levels for Chop/Gadd153 (DNA Damage Inducible Transcript 3), an apoptotic gene activated under endoplasmic reticulum (ER) stress. The role of ER stress in dieldrin-mediated neurotoxicity has been demonstrated nicely in second cell model for PD (cultured dopaminergic SN4741). In that study, 20 μM dieldrin increased the expression of multiple transcripts in the endoplasmic reticulum (ER) stress response such as chaperone GRP78 and heme oxygenase-1 (Park and Chun, 2016). RNA interference was also conducted to block the induction of CHOP, and this action almost completely repressed dieldrin-induced apoptotic cell death, suggesting ER stress is a significant mechanism underlying dieldrin-mediated neurotoxicity. Other studies have shown that dieldrin induces apoptosis in cells via mechanisms involving caspase 3 signaling and protein kinase C delta activation (Kanthasamy et al., 2003, 2008) as well as phosphorylation by the non-receptor tyrosine kinase Fyn (Saminathan et al., 2011). The effects of dieldrin on the expression of the dopamine system are also evident *in vivo*. Female mice administered either 0.3, 1, or 3 mg/kg dieldrin during the gestation period resulted in significant increases in dopamine transporter mRNA and protein in male and female offspring (Richardson et al., 2006). Oxidative stress is also present in mesostriatal regions of the rodent (Hatcher et al., 2007). Thus, dieldrin-induced neurodegeneration involves endoplasmic reticulum stress, mitochondrial dysfunction and oxidative stress, and apoptosis in both cell and animal models.

Large-scale omics-based investigations have also been conducted with dieldrin to identify the scope of molecular pathways perturbed by the pesticide. Cowie and colleagues fed dieldrin to male and female adult zebrafish for 21 days, using an exposure regime that resulted in body burden levels similar to those in fish collected from polluted environments (Cowie et al., 2017a,b). The study conducted transcriptomics in the CNS of female zebrafish, identifying transcriptional responses that indicated mitochondrial dysfunction, inflammation, and immunosuppression. Quantitative proteomics studies also revealed that dieldrin affects the abundance of proteins related to mitochondrial dysfunction (e.g., ATPase synthase, NADH dehydrogenases), perhaps indicative of compensatory responses in the CNS. Proteins associated with oxidative stress were also differentially expressed in the hypothalamus of zebrafish, further supporting the hypothesis that dieldrin induces mitochondrial dysfunction *in vivo*. Proteomics and transcriptomics studies have also been conducted in apex predators such as largemouth bass, the significance being that this predator must cope with potentially high body burdens in polluted environments over long periods. In a sub chronic feeding study, largemouth bass were fed 2.95 mg dieldrin/kg feed for approximately 2 months (Martyniuk et al., 2010). Transcriptomics and proteomics revealed that molecules related to oxidative stress response, nucleotide base excision repair, response to toxin, and metabolic processes were affected with exposure. Moreover, proteins associated with neurodegeneration (e.g., apolipoprotein E, microtubule-associated tau protein, enolase 1) were altered in abundance by dieldrin in the CNS. In another study, male and female largemouth bass were fed food pellets that were contaminated with 3 mg dieldrin/kg feed, and the exposure regime was again designed to mimic environmental exposure (Martyniuk et al., 2013). Transcriptomics in the male and a female hypothalamus revealed differences between the sexes in response and few gene regulatory pathways overlapped between the sexes. Most striking was that the majority of cell pathways identified by the gene set enrichment were significantly increased in females while the majority of cell pathways were significantly decreased in males fed dieldrin. In the subnetwork enrichment analysis, genes related to dopamine receptor DRD1 and DRD2 signaling were significantly affected (DRD1 network was increased in females with dieldrin while DRD2 signaling network was down-regulated in males), as well as those related to GABAA receptor and retinoic acid receptor signaling. Based on omics approaches with dieldrin and animal models, it appears as though this pesticide alters the expression of genes and proteins associated with neurodegeneration, mitochondrial function, oxidative stress responses, and neurotransmitter signaling.

### Paraquat

#### Brief History of Use

Paraquat or methyl viologen was synthesized for the first time in 1882 and further developed by the Imperial Chemical Company in 1958 after discoveries were made regarding its herbicidal properties. The herbicide was then marketed for agricultural purposes for the first time in 1962 in the United Kingdom (US EPA, 1997). Currently, the world biggest manufacturer of paraquat is Syngenta, with its China plant selling approximately 109,000 tons/year with exports reaching 53,000 tons in 2009 (Watts, 2011). In the US in 2012, the EPA reported that 2–6 millions of pounds of paraquat were used for agricultural purposes (Atwood and Paisley-Jones, 2017). Nonetheless, despite its effectiveness as an herbicide, paraquat has been banned in 32 countries due to emerging evidence for organ toxicity (i.e., paraquat lung) (Gawarammana and Buckley, 2011), association to PD (Tanner et al., 2011), and uncertainty about other adverse potential effects to animals and humans. In the US, paraquat can only be sprayed under the supervision of a certified applicator and it is prohibited to be used in homes, schools, golf courses, recreational parks and playgrounds (US EPA, 1997; EPA, 2011).

Paraquat is used as a defoliant and plant growth regulator, in order to control grasses and broadleaf weeds in over 100 different crops (Paraquat Information Center on behalf of Syngenta Crop Protection AG, 2010)^1^. Between 1995 and 2001, 3.9% of the total paraquat sales were used in oil palm plantation and 2.5% in tea estates (Gochez, 2009). Other crops for which paraquat is used include orchards, soybeans, tice, cotton, wheat, coffee, vegetables, maize, apples, cocoa, and oranges among others. Paraquat is used as a pre-harvest desiccant on cereals, cotton, pineapple and sunflowers; and as post-harvest desiccant to rush removal of spent plants (plants that have been harvested).

#### Mechanism of Action

Paraquat is a non-selective pesticide and is rapidly absorbed by the foliage, essentially rupturing the cell membrane by disrupting photosynthesis. More specifically, paraquat accepts electrons from photosystem I and is then reduced to form the paraquat radical, which reduces molecular oxygen to form superoxide radical. This radical reacts with itself in the presence of superoxide dismutase to form hydrogen peroxide. Hydrogen peroxide and superoxide radicals react to generate the hydroxyl radical, which is extremely reactive and rapidly destroys unsaturated lipids, including membrane fatty acids and chlorophyll. The hydroxyl radical produces lipid radicals that react with oxygen to form lipid hydroperoxides, which in turn destroy the integrity of the cell membrane. This allows the cytoplasm to leak into the intercellular space, resulting in rapid leaf wilting and desiccation. The superoxide radical and hydrogen peroxide can potentially oxidize thiol groups on many proteins within the cell and can become sequestered in different parts of the plant causing damage (Ditomaso et al., 1993; Ranjbar, 2014). Thus the potential for unwanted redox reactions is high with the herbicide, a mechanism that can contribute to the adverse effects observed in non-target organisms.

Due to the high capacity of paraquat to mediate redox reactions, toxicity in mammals causes damage to proteins and lipids in the mitochondria via the production of free radicals and oxidative stress. Studies show that complex I, also known as NADH: ubiquinone oxidoreductase, of the electron transport chain is the main site of superoxide production enabled by paraquat (Cochemé and Murphy, 2008), leading to apoptosis. This is because paraquat is a powerful redox cycler, and the paraquat dication accepts one electron to form the paraquat monocation radical which reacts with oxygen to form superoxide. In biological systems, enzymes containing flavin oxidoreductase use NAD(P)H as electron donors, which facilitates the ability of paraquat to further damage tissues and cells (Cochemé and Murphy, 2008). Other sites in the mitochondria are also reported to be targets for paraquat dication reduction, including NADH-cytochrome b_5_ oxidoreductase and NADH-coenzyme Q oxidoreductase of the mitochondrial outer membrane (Hirai et al., 1992; Shimada et al., 2009).

#### Epidemiological Evidence

Paraquat as mentioned above, is an effective herbicide that can be safely used to some extent when recommendations are adhered too. However, over the years, numerous fatalities have been recorded due to accidental or voluntary ingestion, preceded by clinical signs that include nausea, irritation, diarrhea, pulmonary fibrosis, acute tubular necrosis and pulmonary hemorrhage (Dinis-Oliveira et al., 2008). Epidemiological data suggest a strong association between paraquat and Parkinson Disease (PD). Several epidemiological studies have suggested this correlation between the incidence of random PD and environmental exposure to paraquat (Table 2). For example, a cohort study by Engel et al. (2001) reported that after examining 310 subjects, 17.5% of those were exposed to paraquat and had a parkinsonism prevalence ratio of 0.8 (0.5–1.4 95% Confidence Interval). Another cohort study by Kamel et al. (2007) reported that 20% of cases of those exposed to paraquat had an odds risk ratio of 1.8 with a 95% CI of 10 to 3.5. These cases were considered prevalent PD (i.e. genetic), while the incidental PD (i.e., sporadic) was seen in 16% of the cases with an odds risk ratio of 1.0 and a 95% CI of 0.5 to 1.9. There are a number of case-control epidemiological studies associating paraquat with increased risk to sporadic PD (Table 2). Most recently, it was found that 81 cases out of 362 had a 1.26 OR (0.86, 1.86 95% CI); these 81 subjects had occupational contact with paraquat. The authors also found that 109 cases had residential exposure, with an OR of 0.91 (0.63, 1.31 95% CI) (Wang et al., 2011). In another study, Tanner et al. (2011) evaluated the risk of exposure to several pesticides, including paraquat. The study found that 24% of the subjects showed an association between PD and paraquat exposure with a 2.5 OR (1.4–4.7 95% CI, *p*-value = 0.004). Compared to those individuals that never used or were knowingly exposed to paraquat, the associations with PD were stronger among those who had used pesticides (paraquat and rotenone more specifically). The study also found that PD was diagnosed at a younger age in the group exposed to paraquat. Although confounding variables such as chemical mixtures, age and smoking can confound strength of the correlation between paraquat and PD in human populations (Hertzman et al., 1990, 1994), many studies contain an adequate subject pool size and sufficient details on exposure history. Thus, despite differences in study design and populations investigated, epidemiological studies provide reasonably strong evidence that paraquat plays a role in sporadic PD.

#### Cellular and Molecular Responses

Cell and animal models for PD have been essential for understanding PD etiology, and critical for elucidating mechanisms of pesticide-associated PD. In the 1980's, hypotheses emerged that paraquat negatively impacted the dopaminergic system and therefore, directly contributed to PD pathology. This notion was further strengthened upon the discovery that the structure of paraquat resembled that of 1-methyl-4-phenyl-1,2,3,6-tetrahydropyridine or MPTP, a neurotoxin that is actively shuttled into dopaminergic neurons by the dopamine active transporter. Following this revelation, nigrostriatal degeneration of dopamine neurons by paraquat has been observed in animal models. The literature contains an array of studies that establish mechanisms of paraquat neurotoxicity. Here we give but a few examples from cell and animal models, demonstrating mechanistic and clinical similarities between paraquat and PD.

There are a number of studies in cells showing that paraquat can modulate dopamine synthesis and induce apoptosis. For example, paraquat increases the levels of cytosolic and vesicular dopamine, and can also increase transient TH activity in rat adrenal pheochromocytoma PC12 cells (Izumi et al., 2014). This effect is also associated with the formation of quinoproteins at 50 μM paraquat. Izumi and co-authors suggested that the vulnerability of dopaminergic cells is attributable to endogenous dopamine-stress such as the increase in cytosolic dopamine, which is caused by an increase in TH synthesis and subsequent quinoprotein formation by paraquat. Another study in PC12 cells showed that treatment with paraquat and a methyltransferase inhibitor (5 -aza-2-deoxycytidi) for 12 and 24 h respectively, significantly decreased cell activity and increased apoptosis, increasing cytochrome C and Bax expression and suppressing Bcl2 expression. The results of this study suggest that co-exposure of 5 -aza-2-deoxycytidi (which modulates DNA methylation) and paraquat induces a higher degree of toxicity on PC12 cell by increasing oxidative stress and mitochondria dysfunction. In whole animals, McCormack et al. (2002) demonstrated that, after exposing male C57BL/6 mice to one dose of either 1, 5 or 10 mg/kg paraquat dichloride hydrate (mice ages ranged from 6 weeks, 8 weeks, 6 months to 18 months old) for 3 weeks, dopaminergic neurons were destroyed in the substantia nigra (SN) pars compacta, determined by stereological counting of tyrosine hydroxylase (TH)- immunoreactive and Nissl-stained neurons. The severity of the response was dose and age dependent. The authors also observed degenerating cell bodies by silver staining in the substantia nigra (SN) pars compacta and an enhancement of dopamine synthesis, suggested to be because of increased tyrosine hydroxylase (TH) activity. Other studies have shown that chronic, low-dose systemic exposure to paraquat mimics clinical signs similar to PD including reduced motor function (Thiruchelvam et al., 2003). Thus, paraquat causes selective dopamine degeneration which is associated with changes in locomotor function, both hallmarks of PD.

Computational toxicogenomics has furthered our understanding of paraquat toxicosis, but while studies have established correlations between paraquat and neurodegenerative diseases, the etiology of sporadic forms of age-related diseases and the mechanisms involved are still largely unknown. Recently, Jafari et al. (2017) (Jafari et al., 2017) showed that paraquat preferentially binds to A-T rich regions of DNA in the minor groove using fluorescent spectra in MCF-7 cells, demonstrating that paraquat can act via genotoxic mechanisms. Jafari et al. (2017) used computational approaches to show that the most likely mode of action of paraquat as a DNA binding agent was via an intercalation of 4-pyridyl rings between AT base pairs. In addition to direct effects on the DNA, transcriptional machinery and gene expression can me modulated by paraquat. For example, paraquat has also been shown to sensitize dopaminergic neurons, which leads to the temporal silencing of Pink1 gene expression. This event leads to a significant loss of dopaminergic neurons (Zhou et al., 2011). Other studies have linked paraquat exposure to increased expression of genes such as dopamine transporter (DAT), cytochrome P450 family 2 subfamily E member 1 (CYP2E1), glutathione S-transferase alpha 4 (GSTA4-4), Metallothionein-1 (MT-I) and Metallothionein-2 (MT-II) (Kumar et al., 2010) supporting the hypothesis that paraquat induces neurodegeneration via oxidative stress. Morán et al. (2008) exposed neuroblastoma SH-SY5Y cells to 1–25 μm paraquat and conducted transcriptomics, finding that paraquat induced differential expression of Bik, Cradd, Dapk, Fas, Casp3, and Casp7 in addition to other related transcripts. Another omics-based study focused on gene expression patterns in mice exposed to both maneb (30 mg/kg) + paraquat (10 mg/kg); the study showed that there was a time-dependent alteration in the expression of transcripts associated with several pathways including dopamine synthesis (TH), and antioxidant defense machinery in the mouse striatum (Patel et al., 2008). Downregulation of hexokinase-I (after 6- and 9-weeks of exposure), aconitase-2 (after 9-weeks of exposure), cytochrome C1 (9-weeks of exposure), and cytochrome-C oxidase (9 weeks of exposure) were reported in the study. Studies are not limited to transcripts for paraquat, and now efforts have turned to other regulators of the transcriptome. Wang et al. (2018) studied the effects of paraquat in miRNA regulation in neuro-2a cells (Wang et al., 2018). After treatment with 300 μm paraquat, the authors reported that there was miRNA dysregulation, and up to 228 miRNAs were downregulated and 60 miRNAs were upregulated following exposure to paraquat. The authors also found alterations in the expression of miR-210-3p, miR-374-5p, miR-503-5p, miR-378-3p. Moreover, the overexpression of miR-17-5p suppressed apoptosis, enhanced cell proliferation and promoted S phase transition of the cell cycle. Using the latest techniques in genetic screening, Reczek et al. (2017) discovered through a CRISPR-based positive-selection screen that three specific genes, ATP7A (copper transporter), SLC45A4 (sucrose transporter), and POR (cytochrome P450 oxidoreductase) are necessary for paraquat induced cell death; but more importantly they discovered that POR is the key gene for ROS production induced by paraquat toxicity (Reczek et al., 2017). Thus, new screening approaches are generating exciting insight into the genes associated with paraquat-induced PD.

Studies have also examined the gene-metabolome response to paraquat toxicity. Roede et al. (2011) used a combination of transcriptomics and metabolomics to investigate the mechanisms of toxicity of paraquat and maneb co-exposure; the authors measured over 30,869 transcripts and 1358 metabolites and demonstrated that paraquat and maneb caused more damage when combined as opposed to when they were administered as single treatments (Roede et al., 2014). The transcriptome–metabolome-wide association study (TMWAS) mapped 4 clusters of genes and associated metabolites. One cluster contained genes for two cation transporters and a cation transporter regulatory protein (also a pro-apoptotic protein) while other clusters uncovered stress response genes and transporters linked to cyto-protective mechanisms and cell proliferation. In another study investigating the metabolomics of paraquat, it was observed that the energetic pathways through which paraquat affected the mitochondria included alteration in the phosphate pathway (PPP) metabolome, and this was accompanied by increased concentrations of erythrose-4-phosphate, fructose-6-phosphate, glucono-1,5-lactone, glucose-6- phosphate (Lei et al., 2014). This was also accompanied with an inhibition of glycolysis and the TCA cycle. The study also conducted proteomics, reporting that an overexpression of G6PD increased paraquat induced cell death. Therefore, the hijacking of PPP by paraquat contributes to its redox cycling and subsequent cell death. In summary, there are a wide array of examples from literature that use “Toxico-omics” approaches to uncover novel mechanisms of paraquat-induced neurotoxicity, and this is expected to continue as new high throughput screening approaches emerge in cell and animal models.

### Rotenone

#### Brief History of Use

Rotenone was used as a piscicide began in the United states in the 1930s (Dawson et al., 1983), but as early as 1848, derris root extracts containing rotenone were used by gardeners in the East Indies (La Forge et al., 1933). Even before this, plants containing rotenone were used as a piscicide for fishing in South America (Robertson and Smith-Vaniz, 2008). Rotenone is a naturally-occurring lipophilic isoflavonoid compound that is found in various plants in the Leguminosa family. Rotenone was voluntarily canceled for livestock, pet, residential, and all other uses except as a piscicide in the US and Canada in 2006 and 2008 respectively (Pest Management Regulatory Agency-Canada, 2008; EPA, 2015). This predominant use of rotenone to eradicate undesirable fish species or in sampling fish populations is assumed to be relatively safe due to the low half-life of rotenone in waterways and low absorption across the gastrointestinal tract (Ott, 2006; Robertson and Smith-Vaniz, 2008). However, there is still some concern as to the environmental (particularly to benthic organisms) and public health effects associated with rotenone. Rotenone containing products continue to be used globally as an insecticide in some countries and additional studies of the long term health impacts of rotenone exposure are warranted.

#### Mechanism of Action

Rotenone is a potent semiquinone-antagonist type inhibitor of complex I (NADH: ubiquinone oxidoreductase) of the electron transport chain at a region of the ubiquinone pocket with a partially overlapping binding site of both type I/A and type C inhibitors (Degli Esposti, 1998; Okun et al., 1999). This inhibition generates reactive oxygen species that are capable of damaging proteins, nucleic acids, and lipids in both the mitochondria and cell, leading to apoptosis. This is specifically damaging in the CNS due to low antioxidant activity, high oxygen consumption, and the presence of oxidizable polyunsaturated fatty acids (Zhou et al., 2016). Rotenone-induced inhibition of complex I can also lead to ATP depletion which has been proposed as one of the mechanisms rotenone toxicity (Sherer et al., 2003). In addition to ROS generation, rotenone has been shown to independently inhibit cell proliferation during microtubule assembly by binding to tubulin (Brinkley et al., 1974; Marshall and Himes, 1978; Srivastava and Panda, 2007).

####  Epidemiological Evidence

Rotenone is a selective non-systemic botanical insecticide whose use has been severely restricted due to its neurodegenerative capabilities and association to PD (EPA, 2015). Selective loss of dopaminergic neurons in the substantia nigra are one of the hallmark causes of motor deficits associated with PD (Betarbet et al., 2000) along with the formation of Lewy bodies in the surviving SN neurons (Johnson and Bobrovskaya, 2015). Epidemiological studies have correlated rotenone exposure alone or in combination with other pesticides with an increased risk of developing PD in humans (Dhillon et al., 2008; Hancock et al., 2008; Tanner et al., 2011; Furlong et al., 2015; Pouchieu et al., 2017) (Table 2). In one case-control study, mitochondrial complex I inhibiting pesticides (OR = 1.7 [95% CI, 1.0–2.8]) and particularly rotenone (OR = 2.5 [95% CI, 1.3–4.7]) were associated with an increased risk of developing PD, as well as chemicals that induce oxidative damage (OR = 2.0 [95% CI, 1.2–3.6]), for example paraquat (OR = 2.5 [95% CI, 1.4–4.7]) (Tanner et al., 2011). Another case control study assessing protective glove use found an association of rotenone use and PD with or without glove use (OR = 5.5 [95% CI 1.1, 27.1]), though two or more safety practices lowered this risk (Furlong et al., 2015). Additionally, glove use modified incidence of PD for paraquat (OR = 3.9 [95% CI 1.3, 11.7]) and permethrin (OR = 4.3 [95% CI 1.2, 15.6]) (Furlong et al., 2015). Lastly, a recent cohort study of 181,842 agricultural workers (of which 4916 were exposed to rotenone) also detected an increased likelihood of developing PD with rotenone exposure (OR = 1.57 [95% CI 1.08, 2.29), especially with prolonged exposure to the pesticide (Pouchieu et al., 2017).

#### Cellular and Molecular Responses

Parkinson's is a complex disease with both genetic and environmental components, and the rotenone model of PD has uncovered many candidate genes and networks that could underlie the link to PD (Klemann et al., 2017). The suspected mechanisms of rotenone induced DA cell loss include loss of mitochondrial function, apoptotic pathways, oxidative stress, inflammation, microglial activation, protein aggregation and degradation, glutamate excitotoxicity, depletion of neurotrophic factors, gene expression changes, calcium elevation, cell cycle activation, inhibition of neural stem cell migration and proliferation, activation of the p38 and JNK pathways, among others (Xiong et al., 2012). Dopamine levels also appear to confer sensitivity to rotenone exposure (Watabe and Nakaki, 2007; Abdin and Hamouda, 2008), potentially because ROS are generated during dopamine metabolism (Sherer et al., 2003). Several studies altering α-synuclein prior to rotenone insult suggest that it sensitizes dopaminergic neurons to rotenone induced mitochondrial impairment (Cannon et al., 2009; Mulcahy et al., 2013). An experiment in nematodes suggested that leucine rich repeat kinase (*Lrrk*) offers protection against rotenone induced DA cell loss, and that RGS2 (Regulator Of G Protein Signaling 2) modulates pathway activities between LRRK2 and the PD-associated genes, PINK1 and DJ-1 (Dusonchet et al., 2014). In particular, this study created a transcriptional network for LRRK2 and used an RNAi approach to test 687 putative LRRK2 interactors for neuroprotective effects to rotenone, finding that 280 of the 687 tested genes influenced the LRRK2-mediated DA survival to the chemical. These included known Parkinson's genes (e.g. PRKN, PINK1, and DJ-1), as well as known LRRK2-associated genes (e.g., TUBB, FAS, and members of the WNT and MAPK signaling pathways), and novel regulators of LRRK2 with many downstream neighbors, such as Actin (ACTA1) and two Wnt signaling members (i.e. FZD1 and TNKS) (Dusonchet et al., 2014). Another study gathered transcriptome data for SH-SY5Y neuroblastoma cells exposed to 5 nM or 50 nM rotenone for 1 and 4 weeks, and found a total of 134 genes (40% commonly affected) at 1 week and 825 genes (30% commonly affected) at 4 weeks, associated to apoptosis, proliferation, cell cycle, DNA damage response, transcription, and differentiation (Cabeza-Arvelaiz and Schiestl, 2012). Of note, this study also found transcriptional changes to microtubule assembly that could promote mitotic arrest and genomic instability, specifically downregulation of TUBB3, TUBB4, and TUBB6, upregulation of the APC gene, differential expression of CHFR, and suppression of TPPP and CAV1(Cabeza-Arvelaiz and Schiestl, 2012). As such, these toxicogenomics studies have provided new insight into the mechanisms of rotenone-induced neurotoxicity.

### Maneb

#### Brief History of Use

Maneb was first registered as a broad-spectrum pesticide in the United States in 1962 (EPA, 2005). Maneb is a manganese-containing ethylenebis-dithiocarbamate fungicide (EBDCs) (Kanchi et al., 2014). Maneb is widely used as a pesticide on fruit and nut crops, vegetable crops, field and forage crops, grapes, field crop seeds, and other varied grains and legumes (Kapoor et al., 1994; EPA, 2005). Approximately 9 million pounds of the manganese-containing fungicides mancozeb and maneb were applied annually in the U.S. (EPA, 2011), and a significant amount of chemical is released annually into the environment. Maneb and two other DTC fungicides, mancozeb and metiram, are metabolized into ethylene thiourea (ETU) in non-target organisms and eventually degrade to ETU in the environment. Due to potential carcinogenic, developmental, thyroid, and neurotoxic effects on wildlife and humans of the parent compound, as well as risks to human health and the environment that are associated with its metabolites (EPA, 2005), maneb was banned from use by the Swedish Chemicals Agency (SCA) in 2008 (SCA, 2008), the European Parliament (EP) in 2009 (EP, 2009), and in the U.S. by the EPA in 2012 (EPA, 2011). However, the European Union continues to be a major producer and consumer of maneb (epa.gov, 2010; efsa.europa.eu, 2014), and this pesticide is still used in some countries such as the United Kingdom, Austria, Denmark and Spain (Pesticide Properties DataBase for Maneb: http://sitem.herts.ac.uk/aeru/ppdb/en/Reports/426.htm). The total sales of maneb in 2016 were $28 million around the world (http://www.icama.org.cn/hysj/index.jhtml). Thus, concerns remain about the hazards to human health and wildlife.

#### Mechanism of Action

Dithiocarbamates (DTC) are divided into two groups: the ethylenebis-dithiocarbamates (EBDC), such as maneb, zineb, and mancozeb, and the dimethyl-dithiocarbamates (DMDC), including ziram, ferbam, and thiram (Houeto et al., 1995; Kanchi et al., 2014). DTC fungicides can inactivate SH groups in amino acids, proteins and enzymes of the fungi to prevent or inhibit plant diseases (Matheron, 2001). Maneb can attach to SH site of the target fungi through its main active element of manganese ethylene-bis-dithiocarbamate (Mn-EBDC) (Matheron, 2001; Zhang et al., 2003).

#### Epidemiological Evidence

Maneb is a non-systemic and contact fungicide, which leads to the possibility of industrial and agricultural worker exposure (EPA, 2005). Clinical signs upon contact with high doses of maneb include vomiting, diarrhea, headache, confusion, drowsiness, coma, slowed reflexes, respiratory paralysis, and death (Gosselin et al., 1984). To investigate the potential relationship between maneb and PD, Costello et al. (2009) investigated 368 individuals with confirmed PD as well as 341 non-PD (control) from the central valley of California who were expected to be exposed to maneb and paraquat between 1974 and 1999 (Table 2). Their results showed that co-exposure to both pesticides within 500 m of the home increased the risk of PD by 1.75-fold, and a much higher risk occurred when persons aged 60 years or more were exposed to either maneb or paraquat alone (odds ratio = 2.27-fold) or to both pesticides in combination (odds ratio = 4.17-fold) (Costello et al., 2009). Wang et al. (2011) enrolled 362 incident PD cases and 341 controls living in the Central Valley of California in a study from 2001 to 2007. Reported PD cases included individuals that were exposed to the pesticides ziram, maneb, and paraquat and the study demonstrated that combined exposure to these three pesticides increased the risk of PD (odds ratio = 3-fold); in addition, combined ambient exposure to maneb and paraquat at both workplaces and residences increased PD risk substantially (Wang et al., 2011). In another study, a total of 202 PD cases and 362 hospital-based controls were recruited in five hospitals, covering four regions of the Netherlands, between 2010 and 2012 (Brouwer et al., 2017). These individuals were ever exposed to maneb within a 50 m distance around their residence and environmental exposure to this pesticide led to the increase of PD risk (odd ratio = 0.97-fold) (Brouwer et al., 2017). Thus, based on epidemiological studies, there is evidence that maneb, alone or in combination, is linked to PD.

#### Cellular and Molecular Responses

Experimental evidence from animal and cell models also suggests that maneb, alone or co-exposure with other herbicides such as paraquat, causes etiology associated neurodegeneration and PD. Maneb had an inhibitory effect on locomotor activity and aggressiveness in male mice following a 24 h injection of 60 and 100 mg/kg, and the herbicide significantly potentiated the duration of catatonia following a 24 h injection at a dose of 30 mg/kg, suggesting that maneb induces a CNS depressant-like effect. This was hypothesized to act via the dopaminergic system (Morato et al., 1989). Maneb also potentiated the neurotoxic effects of MPTP on locomotor activity and catalepsy in mice (Takahashi et al., 1989). Thus, behavioral assays have shed light on the adverse effects due to maneb toxicosis. In another study, maneb resulted in dopaminergic neurodegeneration, evoked substantial dopamine efflux when infused acutely into the striatum, and preferentially inhibited mitochondrial complex III of isolated brain mitochondria in adult male rats following 14-day treatments at doses of 500 μM and 1 mM daily (Zhang et al., 2003). Maneb also induced neurodegeneration and damage in rat dopaminergic cells at 500 and 1000 ng/mL (1.88 and 3.76 μM) in a 24 h period (Barlow et al., 2005). Thus, experimental evidence exists for an association between maneb and processes related to neurodegeneration.

Previous studies have reported that co-exposure to paraquat and maneb may increase the risk of PD in animal models. For example, nigrostriatal degeneration and locomotor impairment were induced by combined exposure to paraquat and maneb in mice (Thiruchelvam et al., 2000a,b). Age-related irreversible progressive nigrostriatal dopaminergic neurotoxicity was induced in different aged male mice following 6 injections (twice a week for 3 weeks) using a mixture of 10 mg/kg paraquat and 30 mg/kg maneb (Thiruchelvam et al., 2003). Developmental exposure to the combination of paraquat and maneb during the postnatal period produced permanent and progressive lesions of the nigrostriatal dopamine system and enhanced adult susceptibility to these pesticides in mice model (Thiruchelvam et al., 2002). In addition, exposure to a combination of paraquat and maneb in mice led to neuronal α-synuclein pathology and α-synuclein-associated mitochondrial degeneration (Norris et al., 2007). In another study, a significant decrease in tyrosine hydroxylase abundance was observed in the striatum of male mice after 7-day injection with a mixture of 10 mg/kg paraquat and 30 mg/kg maneb per day over 7 days (Gollamudi et al., 2012). Correspondingly, co-exposure to 10 mg/kg paraquat and 30 mg/kg maneb induced neurodegeneration through NADPH oxidase-mediated microglial activation in male mice after intraperitoneal injection for consecutively sex weeks (twice per week) (Hou et al., 2017).

Maneb has been used as a model compound for investigations into pesticide-induced PD, and studies using the compound have generated critical insight into the molecular mechanisms underlying the adverse effects of chemicals in the context of neurodegenerative diseases (Franco et al., 2010). Molecular mechanisms related to maneb toxicosis involve oxidative stress and intracellular GSH pools as observed in rat pheochromocytoma cells (PC12), human neuroblastoma cells (SH-SY5Y), and rodent mesencephalic neuronal cells (MES 23.5), respectively (Zhou et al., 2004; Barlow et al., 2005; Roede et al., 2011). Maneb can also induce mitochondrial dysfunction by binding to mitochondrial complex III in male rat (Zhang et al., 2003), and activate Bak-dependent neuronal death (Fei and Ethell, 2008). The mode of action by co-exposure to paraquat and maneb include nigrostriatal degeneration in male mice (Thiruchelvam et al., 2000a,b, 2005), decreased dopamine turnover (Thiruchelvam et al., 2000a). Bax-dependent neuronal apoptosis in human neuroblastoma cells (Fei and Ethell, 2008), as well as α-synuclein aggregates and tyrosine hydroxylase turnover in human neuroblastoma cells (Caputi et al., 2015).

Additionally, transcriptome sequencing (RNA-Seq) has also provides valuable insight into the mechanisms underlying pesticide-induced PD, and recent efforts with transcriptome analysis showed that a mixture of paraquat (10 mg/kg/day) and (30 mg/kg/day) maneb altered axonal guidance signaling, Wnt/b-catenin signaling, IL-6 signaling, ephrin receptor signaling, TGF-b signaling, PPAR signaling and G-protein coupled receptor signaling pathways in the ventral midbrain and striatum in a mouse PD model after a 7-day injection, suggesting that these pathways contribute in some way to the pathogenesis of idiopathic PD (Gollamudi et al., 2012). In another study, the combination of transcriptomics and metabolomics indicated that maneb alone (10.5 and 27 μM) or in a combined treatment with 90 μM paraquat and 10.5 μM maneb for 4 h altered both transcriptional and metabolic machinery associated with oxidative stress and mitochondrial energy metabolism in mouse catecholaminergic neuronal cells (Roede et al., 2014). Thus, new studies are designed to elucidate the cellular mechanisms underlying neurodegeneration, such as mitochondrial dysfunction and ROS activation.

Apart from their role in generating ATP, the four complexes of the electron transport chain are interconnected to many cellular processes, including the Citric Acid Cycle, aging, and apoptosis to name but a few. Not surprisingly, mitochondrial dysfunction is implicated in a variety of diseases, notably cancer and neural disorders (Hong and Pedersen, 2008; Solaini et al., 2011; Bergman and Ben-Shachar, 2016; Gorman et al., 2016). Moreover, mitochondria and its substrates used for OXPHOS are targets of many commercial pesticides (Casida, 2009; Xiong et al., 2015) and therapeutic drugs for a range of conditions (Casademont et al., 2003; Ghanizadeh et al., 2013; Guo et al., 2016). Inhibitors of these complexes have also served a crucial role in research spanning decades about their structure and function (Sharma et al., 2009; Iverson et al., 2012; Xia et al., 2013).

### Ziram

#### History of Use

Ziram [Zinc bis (dimethyldithiocarbamate)] is a broad-spectrum fungicide, which belongs to the dimethyl-dithiocarbamate (DMDCs) fungicides (Houeto et al., 1995; Kanchi et al., 2014). Ziram was first registered in the United States in 1960 for controlling the scab in apples and pears, leaf curl in peaches, as well as anthracnose and early blight in tomatoes (EPA, 2014). Additional uses were added to the label in 1981 for the control of leaf blight and scab in almonds, shot-hole in apricots, brown rot and leaf spot in cherries, scab and anthracnose in pecans and leaf spot, as well as rust and powdery mildew in ornamentals (EPA, 2014). Other registered uses of this pesticide include homeowner application on residential ornamentals as a rabbit repellent and industrial application as a preservative in exterior latex paints and building materials Approximately 2 million pounds of ziram is used annually (EPA, 2015) and it continues to be applied as a pesticide globally, including the United States, Italy, Japan, India, and China, among other countries (PPDB for Ziram: http://sitem.herts.ac.uk/aeru/ppdb/en/Reports/684.htm; http://www.icama.org.cn/hysj/index.jhtml).

#### Mechanism of Action

As one of the dithiocarbamate (DTC) fungicides, ziram contains the zinc and iron complexes as its activity group (Houeto et al., 1995; Kanchi et al., 2014). Ziram can bind to the SH site of amino acids, proteins, and enzymes of the fungi, lead to the inactivation of these biological molecules in the fungi, and control fungal diseases on a wide range of crops (Matheron, 2001; Zhang et al., 2003).

#### Epidemiological Evidence

Several epidemiological studies have suggested associations between the incidence of PD following environmental exposures to the fungicide ziram (Rhodes et al., 2013; Fitzmaurice et al., 2014; Table 2). In a study by Wang et al. (2011), it was reported that younger onset patients exposed to a combination of ziram and paraquat in the workplace experienced a greater risk of PD (odd ratio = 5.98-fold) than those exposed at residences (odd ratio = 2.78-fold) (Wang et al., 2011). Rhodes et al. (2013) recruited idiopathic PD cases (*n* = 360) and population-based controls (*n* = 816) from three counties in California who were suspected to be exposed to ziram from 1974 to 1999. The results showed that exposure to ziram in the residence as well as the workplace increased the risk to PD (odd ratio = 3.3-fold) (Rhodes et al., 2013). Additionally, Fitzmaurice et al. (2014) enrolled 953 PD patients and 816 controls that were suspected to be exposed to ziram within 500 m in 1974–1999 in three rural California counties (Fresno, Tulare, Kern). Noteworthy was that the data indicated that ambient exposure to ziram only in the residence or only at the workplace had no significant effect on the risk to PD (odd ratio = 0.85- and 1.69-fold, respectively), while exposure to ziram at both residence and workplace conditions resulted in a significant increase in PD risk (odd ratio = 3.52-fold) (Fitzmaurice et al., 2014).

#### Cellular and Molecular Responses

Experimental evidence for neurodegeneration induced by ziram has been generated in both cell and animal models. These experimental models include rat neurons cells (Chou et al., 2008; Rinetti and Schweizer, 2010), rat brain NCX3 cells (Jin et al., 2014), Drosophila (Martin et al., 2014, 2016), and zebrafish embryos (Lulla et al., 2016). Experimental and epidemiological studies have suggested that the inhibition of the ubiquitin–proteasome system (UPS) is one potential mechanisms of the pathogenesis of PD caused by ziram. For example, ziram inhibited the active site of the ubiquitin-activating enzyme (E1) in UPS in rat primary hippocampal neurons after 2-h treatment with a dose of 10 μM (Rinetti and Schweizer, 2010). In another study, significant inhibitory effects on proteasome activity were observed after 1-day and 1-week exposure to 1 and 10 μM ziram in SK-N-MC neuroblastoma cells (Wang et al., 2006). In another study, damaged dopaminergic neurons were even induced in rat primary ventral mesencephalic cells by inhibiting the E1 ligase of the ubiquitin-proteasome system after 10-day treatment with a dose of 0.5 μM (Chou et al., 2008). In male Drosophila flies, a model used to assess the behavioral effects of PD, combined exposure to maneb (100 mM) and ziram (200 mM) for 60 days inhibited E1 ubiquitin ligase genes of the ubiquitin-proteasome system, which resulted in DA cell death (Martin et al., 2014). Additionally, an epidemiological study found that ziram inhibited the proteasome activity in PD patients (Rhodes et al., 2013). Thus, ziram appear to significantly perturb the ubiquitin–proteasome system at the transcriptional and enzyme level.

Other proposed mechanisms of this pesticide include aldehyde dehydrogenase (ALDH) inhibition (Fitzmaurice et al., 2014), intraneuronal calcium (Ca^2+^) dysregulation (Jin et al., 2014), overexpression of synuclein (Lulla et al., 2016), and presynaptic disruption of aminergic and glutamatergic nerve terminals (Martin et al., 2016). ALDH is a mitochondrial enzyme that degrades oxidative metabolites of dopaminergic neurons (Doorn et al., 2014). Fitzmaurice et al. (2014) reported that ziram inhibited the activity of ALDH through its metal complex in the neuron of PD patients, and that genetic variation in ALDH2 exacerbated the PD risk in human who were potentially exposed to ziram over a 26-year period. Jin et al. (2014) demonstrated that intraneuronal calcium (Ca^2+^) dysregulation and subsequent neuronal death were caused in rat brain NCX3 cell with a 24-h treatment of 10 mM ziram. Lulla et al. (2016) found that the overexpression of ZF γ-synuclein 1 (γ1) gene and the selective loss of dopaminergic (DA) neurons were induced in zebrafish embryos after 5-day exposure to 50 nM ziram, which was associated with the pathogenesis of PD. Additionally, in a Drosophila model, exposure to 20 μM ziram for 45 min increased exocytosis and inhibited endocytosis at glutamatergic Type I b terminals, while no detectable effect on exocytosis and dramatical inhibition of endocytosis at octopaminergic Type II terminals were observed, suggesting that these different responses caused by ziram might influence the specific sensitivity of aminergic neurons in PD (Martin et al., 2016). It is clear that there is a putative array of mechanisms associated with ziram-induced PD and continued studies are warranted to more fully discern the mechanism of ziram.

## What are the Common Transcriptome Responses Observed With PD-associated Pesticides?

Our next objective was to determine which transcripts are those most likely regulated by these specific PD-associated chemicals. We extracted data from the Comparative Toxicogenomics Database (CTD) (Davis et al., 2009, 2016) to compile information on gene expression responses for each of the pesticides. The language of CTD is a structured hierarchical vocabulary that describes relationships among chemicals, genes, and proteins based upon interaction data (i.e., expression, regulation). Examples of pesticides associated with PD, based upon expression data evidence in the Comparative Toxicogenomics Database (CTD) is presented in Table 3. The inference score reflects the degree of similarity between CTD chemical–gene–disease networks and a higher score indicates a higher likelihood of interconnectivity among entities. Rotenone, not surprising as a mitochondrial toxicant, was ranked 5th of all chemicals, based upon molecular data associating this chemical to PD. Transcriptome profiles generated by rotenone were strongly associated with those observed in studies of PD. As expected, paraquat and maneb were also associated with PD based upon toxicogenomics responses. The table also depicts other pesticides such as permethrin, a pyrethroid insecticide, and chlorpyrifos, an organophosphate pesticide that have suggested links to Parkinson's disease.

**Table 3:** Examples of pesticides associated with Parkinson's disease (MESH: D010300), based upon expression data from chemicals in the Comparative Toxicogenomics Database (CTD).

	**Chemical Name**	**Chemical ID**	**CAS RN**	**Disease Name**	**Disease ID**	**Inference Score**	**Reference Count**	**Rank**
A)	Rotenone	D012402	83-79-4	Parkinson Disease	MESH:D010300	50.1	89	5
	Paraquat	D010269	4685-14-7	Parkinson Disease	MESH:D010300	39.36	89	9
	Maneb	D008344	12427-38-2	Parkinson Disease	MESH:D010300	32.58	53	11
	Dieldrin	D004026	60-57-1	Parkinson Disease	MESH:D010300	15.43	48	30
B)	Permethrin	D026023	52645-53-1	Parkinson Disease	MESH:D010300	32.77	50	10
	Chlorpyrifos	D004390	2921-88-2	Parkinson Disease	MESH:D010300	29.48	57	15
	Diazinon	D003976	333-41-5	Parkinson Disease	MESH:D010300	26.13	44	17
	Ditiocarb	D004050	147-84-2	Parkinson Disease	MESH:D010300	24.51	43	18
	Ziram	D015039	137-30-4	Parkinson Disease	MESH:D010300	7.22	30	45

*Provided in the table are the Chemical Name, ID and CAS number (if available), as well as the inference score and reference count that supports the relationship. A CAS Registry Number is a unique numerical identifier assigned by Chemical Abstracts Service (CAS). Data was accessed July 2018. (A) Pesticides and their rankings highlighted in this review and (B) Other examples of pesticides that are associated to transcriptome profiles generated in PD studies*.

We obtained all of the transcripts affected by dieldrin, paraquat, rotenone, maneb, and ziram in the CTD, and compared each of these datasets to MPTP (1-methyl-4-phenyl-1,2,3,6-tetrahydropyridine). Curated [chemical–gene interactions|chemical–disease|gene–disease] data were retrieved from the Comparative Toxicogenomics Database (CTD), MDI Biological Laboratory, Salisbury Cove, Maine, and NC State University, Raleigh, North Carolina. World Wide Web (URL: http://ctdbase.org/) [07/2018]. MPTP, when converted to MPP+, causes clinical signs related to PD and has been used extensively as a model compound for chemical-induced PD. In order for the transcript to be included in the summary table (Table 4), the gene had to be regulated by 4 out of the 6 chemicals queried. We reasoned that these transcripts represent those most likely associated with pesticide-induced PD. This resulted in 86 transcripts (Table 4), many of which would be expected to be related to pesticide-induced PD. These transcripts include acetylcholinesterase, a number of caspases (Caspase (Casp) 3, Casp9, Casp12) and apoptotic regulators (Bcl-2, cytochrome C) as well as genes involved in the oxidative stress response (glutathione peroxidase 4, glycogen synthase kinase 3 beta, superoxide dismutase 1 and 2). Inflammatory signaling molecules such as interleukin 1 beta, interleukin 6, and NF kappa B also support a role for neuroinflammation in response to pesticide exposures (Hirsch and Hunot, 2009; Qian et al., 2010). Also included in the list are transcripts related to dopamine synthesis and signaling such as tyrosine hydroxylase and dopamine receptor D2 as well as transcripts that associate to Parkinson's disease following genetic mutation (i.e., alpha-synuclein, Park2, and Lrrk2). The fact that transcriptome responses include many genes known to play a role in PD strengthen the mechanistic links between pesticide exposures and PD.

**Table 4:** Lists of genes regulated by dieldrin, paraquat, rotenone, maneb, and ziram were downloaded from the Comparative Toxicogenomics Database, and compared amongst each other and to MPTP (1-methyl-4-phenyl-1,2,3,6-tetrahydropyridine) which converted to MPP+, causes clinical signs related to Parkinson's disease.

**Gene Symbol**	**Target Database**	**Gene Name**
ACHE	ENSG00000087085	acetylcholinesterase (Cartwright blood group) [Source:HGNC Symbol;Acc:HGNC:108]
AIF1	ENSG00000204472	allograft inflammatory factor 1 [Source:HGNC Symbol;Acc:HGNC:352]
AKT1	ENSG00000142208	AKT serine/threonine kinase 1 [Source:HGNC Symbol;Acc:HGNC:391]
ALB	ENSG00000163631	albumin [Source:HGNC Symbol;Acc:HGNC:399]
APAF1	ENSG00000120868	apoptotic peptidase activating factor 1 [Source:HGNC Symbol;Acc:HGNC:576]
BAD	ENSG00000002330	BCL2 associated agonist of cell death [Source:HGNC Symbol;Acc:HGNC:936]
BAK1	ENSG00000030110	BCL2 antagonist/killer 1 [Source:HGNC Symbol;Acc:HGNC:949]
BAX	ENSG00000087088	BCL2 associated X, apoptosis regulator [Source:HGNC Symbol;Acc:HGNC:959]
BCL2	ENSG00000171791	BCL2, apoptosis regulator [Source:HGNC Symbol;Acc:HGNC:990]
BCL2L1	ENSG00000171552	BCL2 like 1 [Source:HGNC Symbol;Acc:HGNC:992]
BDNF	ENSG00000176697	brain derived neurotrophic factor [Source:HGNC Symbol;Acc:HGNC:1033]
BID	ENSG00000015475	BH3 interacting domain death agonist [Source:HGNC Symbol;Acc:HGNC:1050]
BNIP3	ENSG00000176171	BCL2 interacting protein 3 [Source:HGNC Symbol;Acc:HGNC:1084]
CASP1	ENSG00000137752	caspase 1 [Source:HGNC Symbol;Acc:HGNC:1499]
CASP12	ENSG00000204403	caspase 12 (gene/pseudogene) [Source:HGNC Symbol;Acc:HGNC:19004]
CASP3	ENSG00000164305	caspase 3 [Source:HGNC Symbol;Acc:HGNC:1504]
CASP7	ENSG00000165806	caspase 7 [Source:HGNC Symbol;Acc:HGNC:1508]
CASP8	ENSG00000064012	caspase 8 [Source:HGNC Symbol;Acc:HGNC:1509]
CASP9	ENSG00000132906	caspase 9 [Source:HGNC Symbol;Acc:HGNC:1511]
CAT	ENSG00000121691	catalase [Source:HGNC Symbol;Acc:HGNC:1516]
CCL2	ENSG00000108691	C-C motif chemokine ligand 2 [Source:HGNC Symbol;Acc:HGNC:10618]
CCND1	ENSG00000110092	cyclin D1 [Source:HGNC Symbol;Acc:HGNC:1582]
CDKN1A	ENSG00000124762	cyclin dependent kinase inhibitor 1A [Source:HGNC Symbol;Acc:HGNC:1784]
COX5A	ENSG00000178741	cytochrome c oxidase subunit 5A [Source:HGNC Symbol;Acc:HGNC:2267]
COX7A2L	ENSG00000115944	cytochrome c oxidase subunit 7A2 like [Source:HGNC Symbol;Acc:HGNC:2289]
CREB1	ENSG00000118260	cAMP responsive element binding protein 1 [Source:HGNC Symbol;Acc:HGNC:2345]
CTSB	ENSG00000164733	cathepsin B [Source:HGNC Symbol;Acc:HGNC:2527]
CYC1	ENSG00000179091	cytochrome c1 [Source:HGNC Symbol;Acc:HGNC:2579]
CYCS	ENSG00000172115	cytochrome c, somatic [Source:HGNC Symbol;Acc:HGNC:19986]
CYP1A1	ENSG00000140465	cytochrome P450 family 1 subfamily A member 1 [Source:HGNC Symbol;Acc:HGNC:2595]
DDIT3	ENSG00000175197	DNA damage inducible transcript 3 [Source:HGNC Symbol;Acc:HGNC:2726]
DDIT4	ENSG00000168209	DNA damage inducible transcript 4 [Source:HGNC Symbol;Acc:HGNC:24944]
DRD2	ENSG00000149295	dopamine receptor D2 [Source:HGNC Symbol;Acc:HGNC:3023]
E2F1	ENSG00000101412	E2F transcription factor 1 [Source:HGNC Symbol;Acc:HGNC:3113]
ENO1	ENSG00000074800	enolase 1 [Source:HGNC Symbol;Acc:HGNC:3350]
ENO2	ENSG00000111674	enolase 2 [Source:HGNC Symbol;Acc:HGNC:3353]
FOS	ENSG00000170345	Fos proto-oncogene, AP-1 transcription factor subunit [Source:HGNC Symbol;Acc:HGNC:3796]
GAPDH	ENSG00000111640	glyceraldehyde-3-phosphate dehydrogenase [Source:HGNC Symbol;Acc:HGNC:4141]
GCLC	ENSG00000001084	glutamate-cysteine ligase catalytic subunit [Source:HGNC Symbol;Acc:HGNC:4311]
GFAP	ENSG00000131095	glial fibrillary acidic protein [Source:HGNC Symbol;Acc:HGNC:4235]
GPT	ENSG00000167701	glutamic–pyruvic transaminase [Source:HGNC Symbol;Acc:HGNC:4552]
GPX1	ENSG00000233276	glutathione peroxidase 1 [Source:HGNC Symbol;Acc:HGNC:4553]
GPX4	ENSG00000167468	glutathione peroxidase 4 [Source:HGNC Symbol;Acc:HGNC:4556]
GSK3B	ENSG00000082701	glycogen synthase kinase 3 beta [Source:HGNC Symbol;Acc:HGNC:4617]
GSTA4	ENSG00000170899	glutathione S-transferase alpha 4 [Source:HGNC Symbol;Acc:HGNC:4629]
HBA1	ENSG00000188536	hemoglobin subunit alpha 2 [Source:HGNC Symbol;Acc:HGNC:4824]
HBA1	ENSG00000206172	hemoglobin subunit alpha 1 [Source:HGNC Symbol;Acc:HGNC:4823]
HMOX1	ENSG00000100292	heme oxygenase 1 [Source:HGNC Symbol;Acc:HGNC:5013]
HSPA5	ENSG00000044574	heat shock protein family A (Hsp70) member 5 [Source:HGNC Symbol;Acc:HGNC:5238]
IDH3A	ENSG00000166411	isocitrate dehydrogenase 3 (NAD(+)) alpha [Source:HGNC Symbol;Acc:HGNC:5384]
IFNG	ENSG00000111537	interferon gamma [Source:HGNC Symbol;Acc:HGNC:5438]
IGF1	ENSG00000017427	insulin like growth factor 1 [Source:HGNC Symbol;Acc:HGNC:5464]
IL1B	ENSG00000125538	interleukin 1 beta [Source:HGNC Symbol;Acc:HGNC:5992]
IL6	ENSG00000136244	interleukin 6 [Source:HGNC Symbol;Acc:HGNC:6018]
KIF1B	ENSG00000054523	kinesin family member 1B [Source:HGNC Symbol;Acc:HGNC:16636]
LRRK2	ENSG00000188906	leucine rich repeat kinase 2 [Source:HGNC Symbol;Acc:HGNC:18618]
MAP2K1	ENSG00000169032	mitogen-activated protein kinase kinase 1 [Source:HGNC Symbol;Acc:HGNC:6840]
MAPK1	ENSG00000100030	mitogen-activated protein kinase 1 [Source:HGNC Symbol;Acc:HGNC:6871]
MAPK3	ENSG00000102882	mitogen-activated protein kinase 3 [Source:HGNC Symbol;Acc:HGNC:6877]
MAPT	ENSG00000186868	microtubule associated protein tau [Source:HGNC Symbol;Acc:HGNC:6893]
MTOR	ENSG00000198793	mechanistic target of rapamycin kinase [Source:HGNC Symbol;Acc:HGNC:3942]
MYC	ENSG00000136997	MYC proto-oncogene, bHLH transcription factor [Source:HGNC Symbol;Acc:HGNC:7553]
NFE2L2	ENSG00000116044	nuclear factor, erythroid 2 like 2 [Source:HGNC Symbol;Acc:HGNC:7782]
NFKB1	ENSG00000109320	nuclear factor kappa B subunit 1 [Source:HGNC Symbol;Acc:HGNC:7794]
NOS2	ENSG00000007171	nitric oxide synthase 2 [Source:HGNC Symbol;Acc:HGNC:7873]
NTRK2	ENSG00000148053	neurotrophic receptor tyrosine kinase 2 [Source:HGNC Symbol;Acc:HGNC:8032]
ODC1	ENSG00000115758	ornithine decarboxylase 1 [Source:HGNC Symbol;Acc:HGNC:8109]
PARK2	ENSG00000185345	parkin RBR E3 ubiquitin protein ligase [Source:HGNC Symbol;Acc:HGNC:8607]
PIGC	ENSG00000135845	phosphatidylinositol glycan anchor biosynthesis class C [Source:HGNC Symbol;Acc:HGNC:8960]
PRDX2	ENSG00000167815	peroxiredoxin 2 [Source:HGNC Symbol;Acc:HGNC:9353]
PRDX3	ENSG00000165672	peroxiredoxin 3 [Source:HGNC Symbol;Acc:HGNC:9354]
PTGS2	ENSG00000073756	prostaglandin-endoperoxide synthase 2 [Source:HGNC Symbol;Acc:HGNC:9605]
RELA	ENSG00000173039	RELA proto-oncogene, NF-kB subunit [Source:HGNC Symbol;Acc:HGNC:9955]
SLC18A2	ENSG00000165646	solute carrier family 18 member A2 [Source:HGNC Symbol;Acc:HGNC:10935]
SLC6A3	ENSG00000142319	solute carrier family 6 member 3 [Source:HGNC Symbol;Acc:HGNC:11049]
SNCA	ENSG00000145335	synuclein alpha [Source:HGNC Symbol;Acc:HGNC:11138]
SOD1	ENSG00000142168	superoxide dismutase 1 [Source:HGNC Symbol;Acc:HGNC:11179]
SOD2	ENSG00000112096	superoxide dismutase 2 [Source:HGNC Symbol;Acc:HGNC:11180]
TGFB1	ENSG00000105329	transforming growth factor beta 1 [Source:HGNC Symbol;Acc:HGNC:11766]
TH	ENSG00000180176	tyrosine hydroxylase [Source:HGNC Symbol;Acc:HGNC:11782]
TIMP3	ENSG00000100234	TIMP metallopeptidase inhibitor 3 [Source:HGNC Symbol;Acc:HGNC:11822]
TJP1	ENSG00000104067	tight junction protein 1 [Source:HGNC Symbol;Acc:HGNC:11827]
TNF	ENSG00000232810	tumor necrosis factor [Source:HGNC Symbol;Acc:HGNC:11892]
TP53	ENSG00000141510	tumor protein p53 [Source:HGNC Symbol;Acc:HGNC:11998]
TUBA1A	ENSG00000167552	tubulin alpha 1a [Source:HGNC Symbol;Acc:HGNC:20766]
UCHL1	ENSG00000154277	ubiquitin C-terminal hydrolase L1 [Source:HGNC Symbol;Acc:HGNC:12513]
VEGFA	ENSG00000112715	vascular endothelial growth factor A [Source:HGNC Symbol;Acc:HGNC:12680]

*The transcript had to be present in 4 out of the 6 gene lists to be included in the table below. These represent those transcripts that are most likely associated with pesticide-induced PD*.

While many of these transcripts could be expected based upon what is known about PD, there are some novel transcripts associated with pesticide exposures and PD that are revealed (Table 4). These included phosphatidylinositol glycan anchor biosynthesis class C (Pigc), allograft inflammatory factor 1 (Aif1), TIMP metallopeptidase inhibitor 3, and DNA damage inducible transcript 4. Pigc encodes an endoplasmic reticulum associated protein that is involved in glycosylphosphatidylinositol (GPI) lipid anchor biosynthesis and cytoskeleton. Transcriptomics in male Swiss albino mice undergoing a dietary exposure of MPTP (20 mg/kg body weight) once a day for 4 weeks revealed that Pigc is dramatically unregulated in the striatum (14-fold); the expression of which was reduced with the administration of caffeine (Singh et al., 2010). As another example, Aif1 encodes a protein that binds actin and calcium, and studies show that this mitochondrial protein and regulator of apoptosis is initiated during MPTP toxicity and dopaminergic neuronal cell death (Wang et al., 2003). Moreover, caspase-1 regulates DA neuronal death in mice via caspase-7/PARP1/AIF signaling in the pathogenesis of PD (Qiao et al., 2017). Other examples are Timp3, an inhibitor of the matrix metalloproteinases involved in degradation of the extracellular matrix (ECM) which play a role in neurodegenerative diseases (Rosenberg, 2009), as well as DNA damage inducible transcript 4 (DDIT4) which is induced by cellular stress, including ER stress, and one that regulates the mTOR activity. These genes appear to be preferentially regulated in expression by PD-associated pesticides.

**Figure 2:**
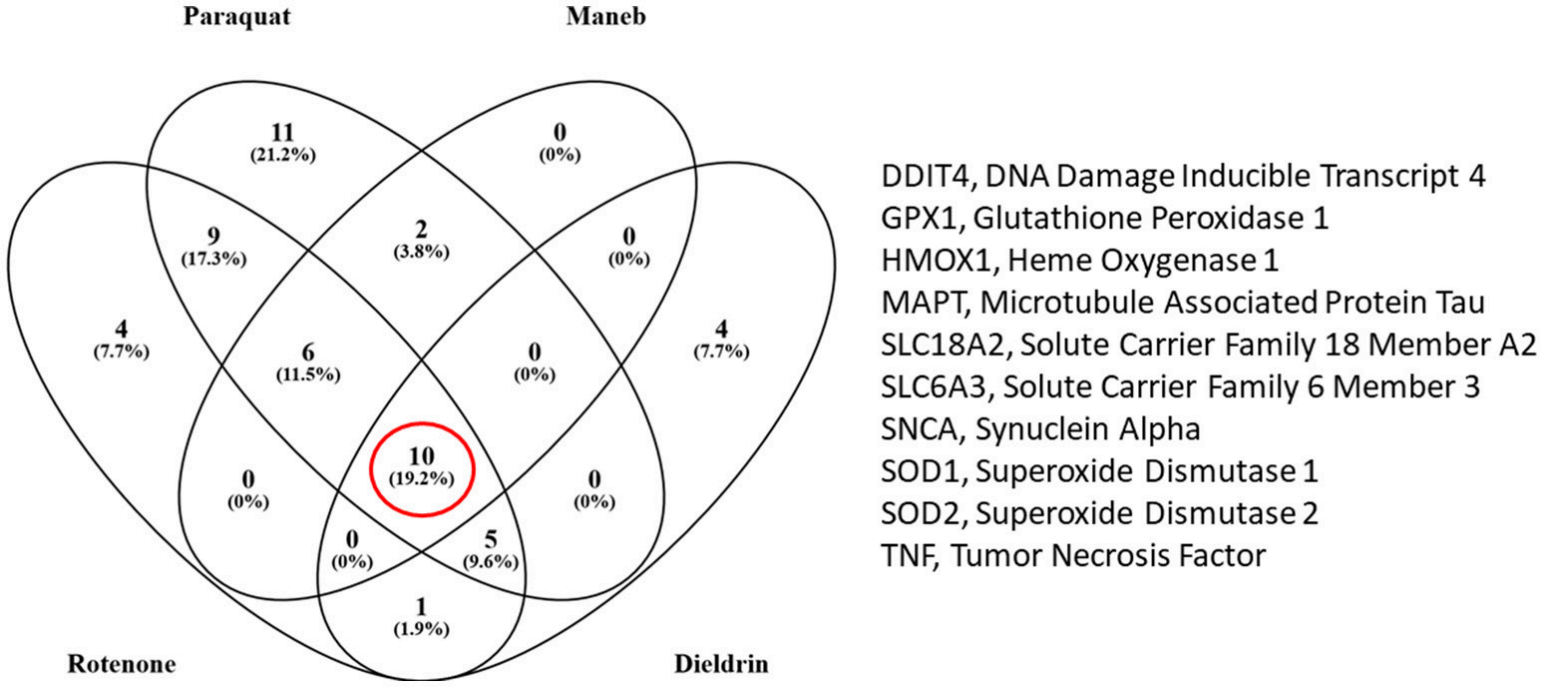
Venn diagram comparing the number of transcripts that were associated with the pesticides under study and Parkinson's disease (PD) (from the Comparative Toxicogenomics Database). There were 10 transcripts that were altered by all four pesticides (rotenone, paraquat, maneb, and dieldrin).

We also extracted all the transcripts that were associated with both the pesticides of interest and PD to determine which transcripts were in common amongst rotenone, paraquat, maneb, and dieldrin. We determined that there were 10 transcripts that were commonly affected by each of these pesticides, all of which are also associated with PD (Figure 2). These included DNA damage inducible transcript 4 (mentioned above) heme oxygenase 1, transcripts related to the oxidative damage response including superoxide dismutase, glutathione peroxidase, vesicular monoamine transporter 2 (Vmat2), dopamine transporter, and transcripts associated with the etiology of PD and neurodegeneration (alpha-synuclein and microtubule associated protein Tau). Thus, the transcriptome responses observed with the exposure to pesticides epidemiologically linked to increased risk to PD include many genes known to be associated to the disease, strengthening the evidence for a direct relationship between exposure and PD manifestation.

**Figure 3:**
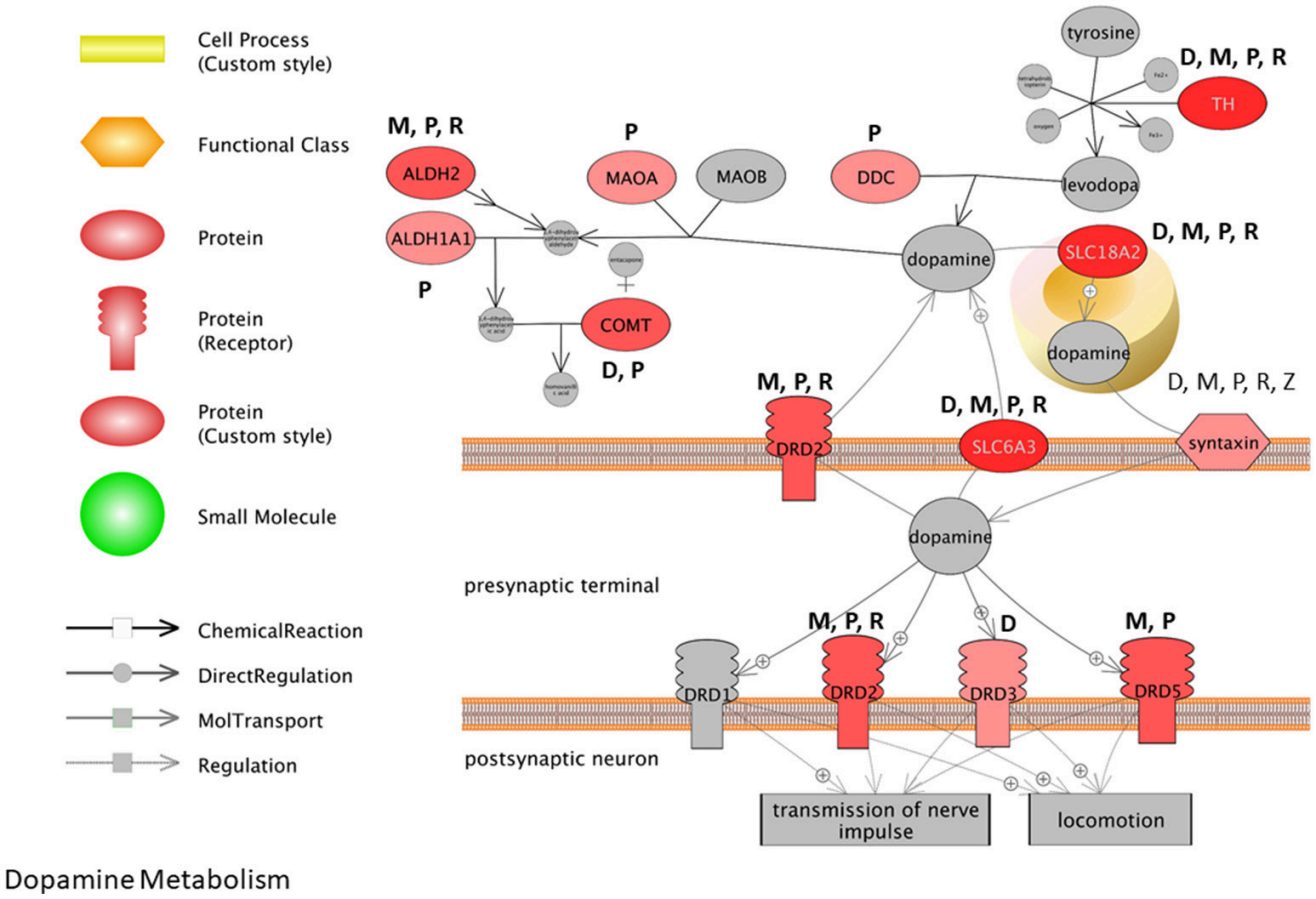
Pathway representation for dopamine metabolism. The darker the red color, the more frequent the association between the gene and the pesticides investigated. M, Maneb; P, Paraquat; R, Rotenone; D, Dieldrin; Z, Ziram.

To visualize how pesticides affect the dopamine synthesis pathway at the transcriptome level, we mapped transcripts to the process of dopamine metabolism. This pathway was extracted from Pathway Studio (Elsevier) and gene expression data for pesticides were overlaid onto the pathway (Figure 3). The darker the red color, the more frequent is the association between the gene and the pesticides investigated here. Syntaxin for example is affected by all 5 pesticides (dieldrin, paraquat, rotenone, maneb, and ziram) while paraquat affects the expression of dopa decarboxylase (Ddc), catechol-O-methyltransferase (Comt), and monoamine oxidase A (Mao-A). Based upon the pathway, it appears as though there are multiple genes involved in dopamine metabolism and signaling that are affected at the expression level by PD-associated pesticides.

Many of the biological themes identified here with pesticides are consistent with transcriptome studies that report on transcripts and cell pathways altered with MPTP-treatments (Bao et al., 2017) and in PD patients compared to health individuals (Gollamudi et al., 2012; Glaab and Schneider, 2015; Mariani et al., 2016). Comparative pathway analysis of the brain transcriptome in PD patients compared to age-matched, non-PD subjects, revealed that gene profiles associated with mitochondria, neuron differentiation, apoptosis, ion channel, and inflammation to name but a few were major entities/processes that were altered with the disease (Glaab and Schneider, 2015). Noteworthy here is that 5 of the 8 GO biological processes identified as having significant associations with the aging- and PD-deregulated genes in the study included synaptic processes, dopamine metabolic process, and dopaminergic synaptic transmission. Moreover, in a recent meta-analysis conducted for transcriptomes of PD patients, it was reported that there were alterations for transcripts related to chaperones, mitochondrial function, and programmed cell death across studies, as well as downregulation of dopamine metabolism pathways (Borrageiro et al., 2018). Based on multiple studies, this has been accompanied with decreased expression of dopamine transporters; solute carrier family 6 member 3 (SLC6A3) and solute carrier family 18 member A2 (SLC18A2) and dopamine decarboxylase. This is consistent with what we observe here with the pesticides under scrutiny, and many genes in the dopamine synthesis pathway are perturbed by these specific chemicals (Figure 3).

**Figure 4:**
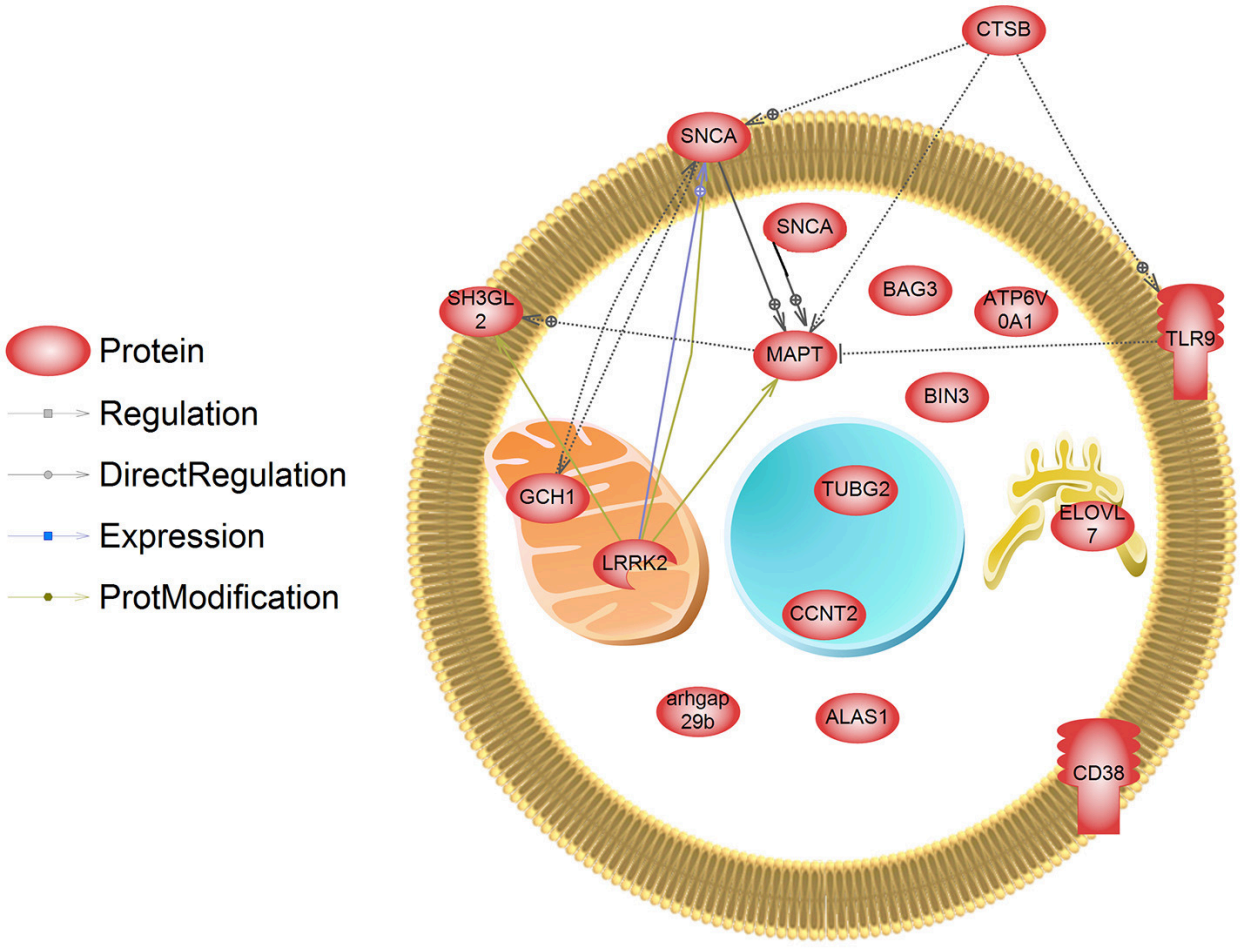
Transcripts that are regulated by pesticides associated with PD. These genes have also been identified in a meta-analysis and GWAS study (Chang et al., 2017) as those containing genetic mutations that are linked to PD. SNCA has been designated as being localized to the both the membrane and cytosol in this depiction, and is a major cytosolic protein but can interact with membranes.

Lastly, we extracted information about genetic loci associated with PD from a recent publication conducting a meta-analysis on the genetics of PD (Chang et al., 2017). Data included information from GWAS and single nucleotide polymorphisms, expression QTL, and transcriptome studies in different animal models for PD (e.g., rodent, Drosophila). We compared these genetic loci to those transcripts that are regulated by PD associated pesticides (collected form CTD). We identified 16 transcripts that are predicted to be genetically linked to PD which are also affected by PD related pesticides (Figure 4, Supplemental Data 1). These transcripts included Bin3, bridging integrator 3 (dieldrin) (proposed to mediate dimerization, sense and induce membrane curvature, and bind small GTPases); Elovl7, Elovl fatty acid elongase 7 (dieldrin) (transferase for fatty Acyl-CoA biosynthesis and metabolism); Ctsb, cathepsin B (dieldrin and rotenone) (lysosomal cysteine protease with endopeptidase and exopeptidase activity, role in protein turnover); and Gch1, GTP cyclohydrolase 1 (rotenone and paraquat) (rate-limiting enzyme in tetrahydrobiopterin (BH4) biosynthesis, catalyzing the conversion of GTP into 7,8-dihydroneopterin triphosphate) among others. These transcripts are hypothesized to play a role in pesticide-induced PD and warrant further study.

## Conclusion

PD is a neurodegenerative disease which is affecting Americans and the global population at a high rate. Environmental factors related to PD remain as important as genetic factors for early onset PD, as there can be significant gene by environment interaction for the disease (Ali et al., 2011). In recognition of this, PD related studies have reported on the transcriptomics, proteomics, and metabolomics responses in the CNS to pesticides. This review provides an overview of the mechanism associated with pesticide-induced neurotoxicity in relation to PD and identifies some conserved transcriptional responses among chemicals. However, there are some limitations of the approach used here. Relationships in the CTD are built based on literature—and studies are sporadic in how they interrogate different cell lines, brain regions, and animal models. Dosing and temporal expression patterns also prove challenging when discerning common mechanisms. While we are confident that we have captured many of the transcripts frequently altered by chemicals that are epidemiologically linked to PD, there is currently not sufficient data in a single cell type or brain region for PD to achieve a higher level of resolution with PD-associated pesticides. Moreover, as pointed out by others (Borrageiro et al., 2018), a lack of compliance with data-access recommendations in transcriptome studies can be problematic, as well as differences in analysis pipelines (fold change cut-offs, false positives). These factors act to confound congruence among studies.

Regardless of some limitations, as computational approaches and analysis software improves, new understanding into how pesticides perturb neuronal cell function, specifically dopamine producing cells, are expected to be revealed. Equally important is how dopamine cells respond in a compensatory manner to chemical insults. Xenobiotic metabolism, oxidative stress response, and DNA repair mechanisms are expected, but additional compensatory responses and protective mechanisms are likely also involved for a coordinated cellular response to chemical insult. Computational toxicology can be used to develop comprehensive transcriptomic biomarker framework for PD, in order to uncover new molecular pathways hypothesized to be important in PD etiology. This review presents new hypothesis about molecular signaling cascades that may underlie PD, which can lead to new putative targets for therapeutic investigations in the CNS. Such as framework can be leveraged in adverse outcome pathways to identify interacting signaling pathways for neurotoxicity, as well as to characterize relative risk of newly developed pesticides. New pesticides can then be further investigated for epidemiological evidence of an association to PD.

## Author Contributions

FC, CS, VP-R, and CM wrote the manuscript. CM edited the manuscript.

### Conflict of Interest Statement

The authors declare that the research was conducted in the absence of any commercial or financial relationships that could be construed as a potential conflict of interest.

## Footnotes

^1^http://paraquat.com/use

## Supplementary Material

Supplemental Data 1 Genes collected from the Comparative Toxicogenomics Database for pesticides of interest. Abbreviations for figures are also found in this document.
